# Application of microRNA and mRNA expression profiling on prognostic biomarker discovery for hepatocellular carcinoma

**DOI:** 10.1186/1471-2164-15-S1-S13

**Published:** 2014-01-24

**Authors:** Lin Wei, Baofeng Lian, Yuannv Zhang, Wei Li, Jianren Gu, Xianghuo He, Lu Xie

**Affiliations:** Shanghai Medical College, Fudan University, Shanghai, 200032 P. R. China; State Key Laboratory of Oncogenes and Related Genes, Shanghai Cancer Institute, Renji Hospital, Shanghai Jiao Tong University School of Medicine, Shanghai, 200032 P. R. China; Shanghai Center for Bioinformation Technology, Shanghai Academy of Science and Technology, Shanghai, 201203 P. R. China; School of Life Sciences and Biotechnology, Shanghai Jiao Tong University, Shanghai, 200240 P. R. China

**Keywords:** Hepatocellular Carcinoma, Gene Expression Profile, Gene Set Enrichment Analysis, Prognosis, microRNA

## Abstract

**Background:**

Hepatocellular carcinoma (HCC) is one of the most highly malignant and lethal cancers of the world. Its pathogenesis has been reported to be multi-factorial, and the molecular carcinogenesis of HCC can not be attributed to just a few individual genes. Based on the microRNA and mRNA expression profiling of normal liver tissues, pericancerous hepatocellular tissues and hepatocellular carcinoma tissues, we attempted to find prognosis related gene sets for HCC patients.

**Results:**

We identified differentially expressed genes (DEG) from three comparisons: Cancer/Normal, Cancer/Pericancerous and Pericancerous/Normal. GSEA (gene set enrichment analysis) were performed. Based on the enriched gene sets of GO terms, pathways and transcription factor targets, it was found that the genome instability and cell proliferation increased while the metabolism and differentiation decreased in HCC tissues. The expression profile of DEGs in each enriched gene set was used to correlate to the postoperative survival time of HCC patients. Nine gene sets were found to prognostic correlation. Furthermore, after substituting DEG-targeting-microRNA for DEG members of each gene set, two gene sets with the microRNA expression profiles were obtained that had prognostic potential.

**Conclusions:**

The malignancy of HCC could be represented by gene sets, and pericancerous liver exhibits important characteristics of liver cancer. The expression level of gene sets not only in HCC but also in the pericancerous liver showed potential for prognosis implying an option for HCC prognosis at an early stage. Additionally, the gene-targeting-microRNA expression profiles also showed prognostic potential, demonstrating that the multi-factorial molecular pathogenesis of HCC is contributed by various genes and microRNAs.

**Electronic supplementary material:**

The online version of this article (doi:10.1186/1471-2164-15-S1-S13) contains supplementary material, which is available to authorized users.

## Background

Hepatocellular carcinoma (HCC), is the sixth most prevalent cancer and the third most frequent cause of cancer-related death [1]. More than 50% of the world's HCC cases occur in China (age-standardized incidence rate: men, 35.2/100 000; women, 13.3/100 000) [2]. The pathogenesis of HCC has been reported to be multi-factorial [3, 4]. Liver cirrhosis is the most important risk factor for HCC [1], which occurs in 80%-90% of HCC patients [5]. In China, chronic hepatitis B virus (HBV) infection is another major risk factor [6], which occurs in approximately 85% of HCC patients [7]. Additionally, the great majority of HBV-infected HCC patients (70% and 90%) have coexisting cirrhosis [2].

The complex process of molecular pathogenesis in HCC also indicates that it is caused by multiple types of genes during its development and progression. For years, the combination of microarray and bioinformatics analytical tools have been widely used to find differentially expressed genes in hepatocellular carcinoma and to find differential diagnostic and prognostic markers [8–15]. Many such studies have used pericancerous liver tissue (assumed to be normal) as the control when selecting differentially changed genes in HCC [8–13]. However, because most pericancerous tissue of HCC is cirrhotic, this assumption could miss important basal molecular changes in the cancer microenvironment. Scientists also attempted to look for differentially expressed genes for prognosis in cirrhosis [15] and non-cancerous liver tissues [14]. As we and other researchers have discovered, dynamic dysregulation exists in the development from cirrhosis to HCC [16], and differentially expressed microRNA in peri-cancer has been used for the prognosis of HCC patients [17].

The low survival rate of HCC patients is largely attributed to the high metastasis rate of HCC. Early studies showed that molecular changes in primary HCC tissue already implied future distant metastasis potential [13]. Additionally, the metastases were reported to be influenced by liver microenvironment that can be represented by inflammation/immune response-related signatures of differentially expressed genes [14]. It would be very interesting to know what kind of molecular changes in the pericancerous tissue of HCC also bear a prediction potential for survival.

In this work, by applying gene expression profiling in hepatocellular carcinoma and pericancerous hepatocellular tissues from HCC patients and in normal liver tissues from healthy individuals, we made an effort to investigate the functional transition in pericancerous liver and cancer liver in HCC patients. We identified expression-changed genes in pericancerous liver and HCC tissue. Then, we conducted functional enrichment analyses to demonstrate the mechanism causing these transitional molecular changes. Additionally, we checked the relationship between the expression level of differentially expressed members of each gene set and the postoperative survival time of HCC patients. We found nine gene sets to be potential prognostic markers. Furthermore, according to the targeting relationships between genes and microRNAs, we also substituted microRNAs for the gene members of each gene set, and we attempted to predict the prognosis with the expression level of the microRNAs that target differentially expressed members of gene sets. Two prognosis-related microRNA sets were identified.

## Methods

### Ethics statement

All human materials were obtained according to consent regulation and approved by the Ethical Review Committee of the World Health Organization Collaborating Center for Human Products Research (authorized by Shanghai Municipal Government). The individuals in this manuscript have given written informed consent to publish these case details.

### Expression profile of mRNA and microRNA

The expression profiling of mRNA and microRNA were performed on three types of liver tissues: HCC, pericancerous liver and normal liver. Forty-five pairs of homogenous human primary hepatocellular carcinoma and adjacent pericancerous liver tissues were collected from the surgical specimen archives of the Department of Pathology, First Affiliated Hospital of Zhejiang University (Hangzhou City, Zhejiang Province, China) and Qidong Liver Cancer Institute (Qidong City, Jiangsu Province, China). The pericancerous liver tissues were collected three centimeters away from any liver tumor. Phenotypic information was collected from patients' records (Additional file 1). And none of the HCC patients had received chemotherapy prior to surgical operation. Ten normal liver tissues were obtained from persons who died in traffic accidents. All of these tissues were freshly frozen at -80°C and confirmed by a pathologist. In each tissue, the total RNA was extracted by TRIzol reagent (Invitrogen, CA, USA); the gene expression was profiled by CapitalBio Human 22k oligonucleotide microarray ([GEO:GPL5918]); and the microRNA expression was profiled by CapitalBio Mammalian miRNA Array Services V1.0 ([GEO:GPL6542]). The expression profiling by array is deposited in Gene Expression Omnibus (GEO) [18] with the accession numbers [GEO:GSE45114] (mRNA) and [GEO:GSE10694] (microRNA) [17].

### Differentially expressed genes

Differentially expressed genes (DEG) involved in three comparisons (Cancer/Normal, Cancer/Pericancerous and Pericancerous/Normal) were detected by the limma [19, 20] package in Bioconductor [21] with absolute log2-fold-change > 2 and adjusted p-value < 0.001, which was adjusted by Benjamini and Hochberg's method (BH) [22]. These three groups of DEGs (C/N_all, C/P_all and P/N_all) were further separated into smaller groups, up-regulated DEGs and down-regulated DEGs: C/N_up and C/N_down; C/P_up and C/P_down; and P/N_up and P/N_down.

### Gene set enrichment analysis

Gene set enrichment analysis for each group of DEGs was performed by the HTSanalyzeR [23] package in Bioconductor with the collection of annotated gene sets provided by the Molecular Signatures Database [24] (MSigDB v4.0, released Jun 7, 2013, including 10295 records). The MSigDB collects various types of gene set, including seven major collections: c1, chromosome and cytogenetic band; c2, online pathway database, publications in PubMed, and knowledge of domain experts, its CP sub-collection collected 1320 Canonical pathways derived from the pathway databases of BioCarta [25], KEGG [26], PID [27], Reactome [28] and four others (SigmaAldrich [29], Signaling Gateway [30], Signal Transduction KE [31], SuperArray [32]); c3, conserved cis-regulatory motifs, its TFT sub-collection collected 615 gene sets that contain genes sharing a transcription factor binding site defined in the TRANSFAC (version 7.4) database; c4, computational gene sets defined by mining large collections of cancer-oriented microarray data; c5, gene ontology, collected 1454 gene sets derived from the controlled vocabulary of the Gene Ontology (GO) project [33]; c6, oncogenic signatures; and c7, immunologic signatures. Only when the BH-adjusted p-values from a hypergeometric test and Gene Set Enrichment Analysis (GSEA) [24] were both lower than 0.05 was the gene set thought to be significantly enriched with this group of DEGs.

### MicroRNAs that target differentially expressed genes

By the RmiR [34] package in Bioconductor, we obtained the targeting relationships between microRNAs and genes that appear in at least three microRNA target databases from six: miRBase [35], TargetScan [36], miRanda [37], tarBase [38], mirTarget2 [39] and PicTar [40]. Then, we obtained the set of microRNAs that target differentially expressed genes in each gene set.

### Association between gene (or microRNA) expression profile and postoperative survival time

We used either the DEGs in each enriched gene set or the microRNAs that target DEGs in each enriched gene set to comprise a candidate classifier for prognosis. The associations between gene (or microRNA) expression and postoperative survival time were tested by the phenoTest [41] package in Bioconductor. The effects of the gene expression (or microRNA expression) on survival were tested via the Cox proportional hazards model [42] and Kaplan-Meier estimator [43]. Additionally, these associations were validated on two independent data sets: [GEO:GSE14520] [44, 45] (including gene expression profiles of 227 pairs of cancer and pericancerous liver samples, as well as 2 normal liver samples), and the liver hepatocellular carcinoma tumor type from The Cancer Genome Atlas [46] (TCGA LIHC) (including gene and microRNA expression profiled with RNASeq from 27 pairs of cancer and pericancerous liver tissues). The phenotypic information of 227 patients from [GEO:GSE14520] and 27 patients from TCGA LIHC are provided in Additional file 1.

## Results

### Differentially expressed genes

**Figure 1:**
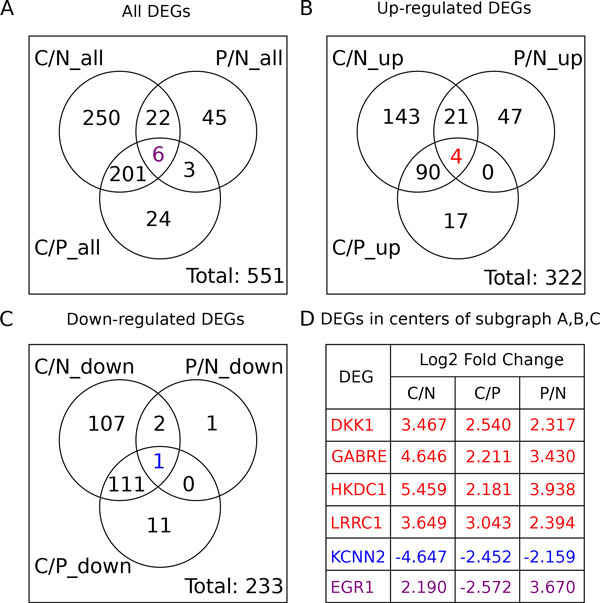
**Venn diagram of differently expressed genes (DEGs) from three comparisons**. Venn diagram of differently expressed genes (DEGs) from three comparisons: Cancer/Normal (C/N), Pericancerous/Normal (P/N) and Cancer/Pericancerous (C/P). A. Venn diagram of all DEGs from three comparisons. The purple number in the center showed the number of DEGs appeared in all three comparisons. B. Venn diagram of up-regulated DEGs from three comparisons. The red number in the center showed the number of DEGs appeared in all three comparisons. C. Venn diagram of down-regulated DEGs from three comparisons. The blue number in the center showed the number of DEGs appeared in all three comparisons. D. The log2-fold-change of DEGs in centers of subgraph A, B and C. The DEGs in red were appeared in the centers of subgraph A and B. The DEGs in blue were appeared in the centers of subgraph A and C. The DEGs in purple was only appeared in the center of subgraph A.

With the threshold of absolute log2-fold-change > 2 and adjusted p-value < 0.001, totally 551 differentially expressed genes (DEG) were identified from three comparisons, Cancer/Normal (C/N, 479 DEGs), Cancer/Pericancerous (C/P, 234 DEGs) and Pericancerous/Normal (P/N, 76 DEGs) (Additional file 2). And subgroups of DEGs from each comparison were selected with up or down regulation of DEGs (Figure 1). In Figure 1, sum of the "up_regulated DEGs" (322) and "down-regulated DEGs" (233) are more than "all DEGs" (555 vs. 551), because some genes were up-regulated in one comparison but down-regulated in another, such as *EGR1* listed in Figure 1D, and they appeared in both Figure 1B and 1C.

Among the 551 DEGs, six genes were differentially expressed in all three comparisons (Figure 1). From Figure 1D, *DKK1, GABRE, HKDC1* and *LRRC1* were up-regulated in pericancerous liver and more up-regulated in cancer liver. The *DKK1* is a Wnt pathway inhibitor, promoting invasion and metastasis of HCC [47], and a serum biomarker for HCC diagnosis [48]. Although the other three DEGs have not been reported in HCC, they are disease related. *GABRE* is related to migraine susceptibility [49]. *HKDC1* is related to Alzheimer disease [50]. And *LRRC1* is DNA repair related [51]. We think they may be important in HCC carcinogenesis. On the contrary, *KCNN2* was down-regulated in pericancerous and more down-regulated in cancer liver. Since *KCNN2* is important for mediating the increase of transepithelial secretion in biliary epithelial cells and prominently expressed in intact liver [52], it seems some function of normal liver was gradually suppressed in pericancerous and cancer liver. And *EGR1* was more up-regulated in pericancerous liver but less up-regulated in cancer liver. Considering that *EGR1* is required for differentiation and mitogenesis [53], the cell proliferation might be up-regulated in both HCC and pericancerous liver, while differentiation might be kept in pericancerous liver but suppressed in HCC.

### Gene sets enriched with differentially expressed genes

Gene set enrichment analysis was performed to identify DEG-related functional gene sets. For each subgroup of DEGs in Figure 1, the gene set enrichment analysis (by hypergeometric test and GSEA) was run on 10295 annotated gene sets in MSigDB v4.0, and a small part of them were enriched with the nine subgroups of DEGs (see the nine circles in Figure 1 A, B and C). The intersections of gene sets enriched with different groups of DEGs were counted in a Venn diagram (Additional file 3). Most gene sets were enriched with both C/N DEGs and C/P DEGs. Especially, the gene sets enriched with both C/N_up DEGs and C/P_up DEGs (or both C/N_down DEGs and C/P_down DEGs) showed the characters present in pericancerous liver but more dys-regulated in HCC. Thus they would provide us some clues about the gradual carcinogenesis of liver tissue.

We further focused on detailed functional analyses of gene sets enriched in three categories of MsigDB v4.0 collection: c5, Gene Ontology (GO) sets; c2, Canonical pathway sets; and c3, transcription factor targets gene sets (TFT). There are 19 GO terms enriched with both C/N_up and C/P_up DEGs (Additional file 4, 5), including biological process (BP) related to "cell cycle" and "mitosis", as well as cellular component (CC) related to "chromosome" and "spindle", showing us the character of cell proliferation that is closely related to carcinogenesis. Meanwhile, 21 GO terms were enriched with both C/N_down and C/P_down DEGs (Additional file 4, 5), including various "metabolism" related BP, CC and MF (molecular function), indicating that metabolisms were disturbed in pericancerous liver and more so in HCC.

Similarly, there are 24 pathways that were enriched with both C/N_up and C/P_up DEGs (Additional file 4, 5). Keywords such as "Cell Cycle", "G1", "S", "G2", "M" and "Replication" indicate the genome instability and cell proliferation hallmark of cancer cells [54] being activated. The "p53" and "p73" related pathways indicate DNA damage and apoptosis found in tumorigenesis. At the same time, the *ATR* (ataxia telangiectasia and Rad3-related [55]) pathway, *PLK1* (polo-like kinase 1 [56]) pathway and the Fanconi anemia pathway showed the ability to repair DNA damage in cancer cells. Thus, as a hallmark of HCC, cell proliferation is the result of rebalancing between active apoptosis by DNA damage and active survival by DNA damage repair. Twenty-one pathways were enriched with both C/N_down and C/P_down DEGs (Additional file 4, 5). The most repetitive keywords are "Metabolism" and "PID_HNF3BPATHWAY" (transcription factor network of *FOXA2* and *FOXA3*), hinting that the function of metabolism regulation and the potential for differentiation were abnormal in HCC, because *FOXA2* (forkhead box A2 [57]) and *FOXA3* (forkhead box A3 [58]) are hepatocyte nuclear factors that act as transcriptional activators for liver-specific genes such as albumin and transthyretin. Similar results have been found in mice [59].

Not only GO and pathway gene sets, but the transcription factor targets gene sets (TFTs) also provided functional annotations for DEGs. We found 19 TFTs were enriched with both C/N_up and C/P_up DEGs (Additional file 4, 5), with the cell cycle controlling transcription factor E2F family being the most conspicuous factor. And *E2F3* and *E2F8* were over-expressed in HCC indeed (Additional file 2). At the same time, only one TFT "RGTTAMWNATT_V$HNF1_01" was enriched with both C/N_down and C/P_down DEGs (Additional file 4, 5).

From the gene ontology, pathway and transcription factor targets related gene sets enriched with both C/N DEGs and C/P DEGs, we found that during cancer progress of HCC, cell proliferation was gradually up-regulated while metabolism was progressively down-regulated. It is rare to observe such phenomena with direct proofs, the advantage stem from our gene expression profiling of gradually changing samples: from normal, to pericancerous, to cancerous liver tissues.

### Association between gene expression profile and postoperative survival time

It is understandable that transitional molecular changes represented by gene sets may demonstrate mechanistic trend of development from normal tissue to cancer tissue, however, whether such changes can be prognostic may be another question.

**Figure 2:**
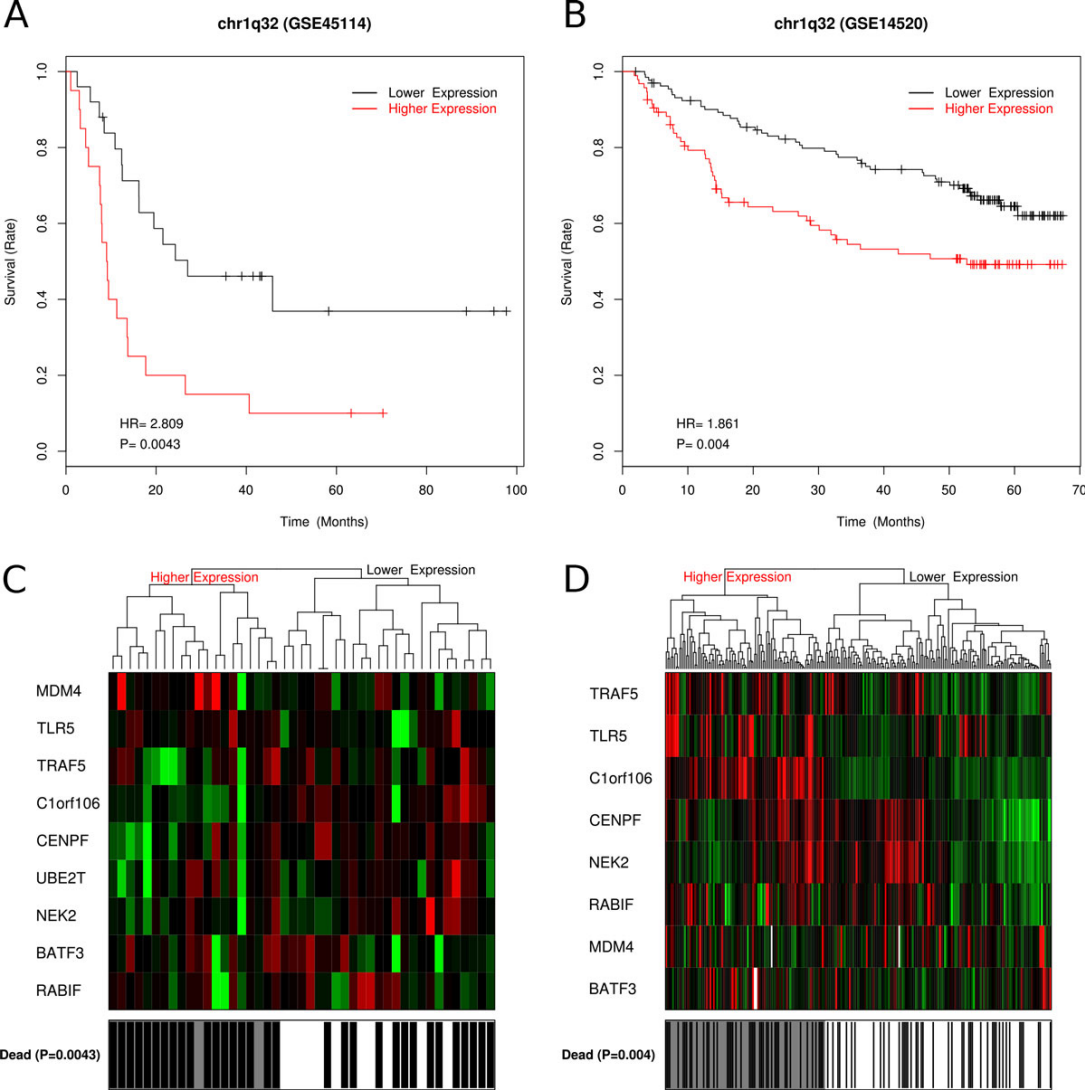
**Prognosis with DEGs in gene set "chr1q32"**. Gene set "chr1q32" could be used for prognosis with DEG members' expression levels in HCC. Kaplan-Meier survival curves and heatmaps of the correlation between the postoperative survival time and the expression profile of differentially expressed gene members in the gene set "chr1q32" with the DEG expression levels in HCC. A. Kaplan-Meier survival curve of DEG expression levels in 45 HCC patients from [GEO:GSE45114]. B. Kaplan-Meier survival curve of DEG expression levels in 227 HCC patients from [GEO:GSE14520]. C. Heatmap of DEG expression levels from [GEO:GSE45114]. D. Heatmap of DEG expression levels from [GEO:GSE14520]. The positive HR (hazard ratio) means the worse prognosis with the higher expression. UBE2T did not appear in subgraph D because this gene was not detected in [GEO:GSE14520]. The remaining DEGs still show significant potential for prognosis.

**Figure 3:**
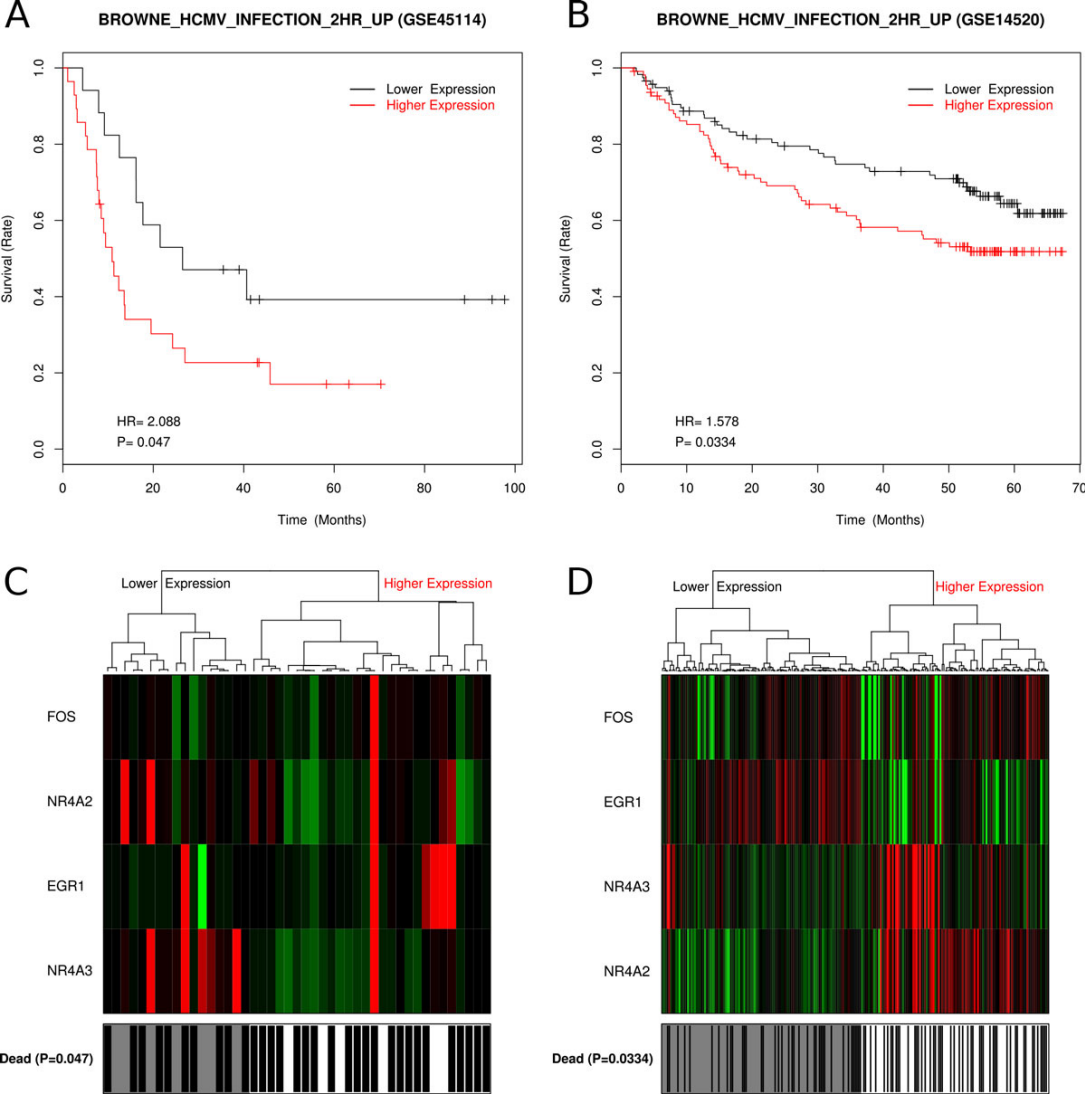
**Prognosis with DEGs in gene set "BROWNE_HCMV_INFECTION_2HR_UP"**. Gene set "BROWNE_HCMV_INFECTION_2HR_UP" could be used for prognosis with DEG members' expression levels in ratio of Pericancerous/Normal. Kaplan-Meier survival curves and heatmaps of the correlation between the postoperative survival time and the expression profile of differentially expressed gene members in the gene set "BROWNE_HCMV_INFECTION_2HR_UP" with the DEG expression levels in the ratio of Pericancerous/Normal. A. Kaplan-Meier survival curve of DEG expression levels in 45 HCC patients from [GEO:GSE45114]. B. Kaplan-Meier survival curve of DEG expression levels in 227 HCC patients from [GEO:GSE14520]. C. Heatmap of DEG expression levels from [GEO:GSE45114]. D. Heatmap of DEG expression levels from [GEO:GSE14520].

The DEGs in each enriched gene set might comprise a candidate gene classifier for prognosis. We tested the association between the expression of these candidate gene classifiers and postoperative survival time in our data set, which was 45 HCC patients from [GEO:GSE45114]. Nine gene sets with the expression level of DEGs that associated with the postoperative survival time in our dataset were also validated in [GEO:GSE14520] (227 HCC patients) (Table 1). As shown in Table 1, Figure 2, 3 and Additional file 6, the expression profile of sets of DEGs in HCC, even pericancerous liver could be used for prognosis.

**Table 1:** Gene sets associated to postoperative survival time with DEG expression profile (validated in [GEO:GSE14520]).

Gene set	Enriched with DEG group	DEG Count	DEG	Profile*
chr1q32	C/N_up	9	BATF3, C1orf106, CENPF, MDM4, NEK2, RABIF, TLR5, TRAF5, UBE2T	C
KAUFFMANN_MELANOMA_RELAPSE_UP	C/N_up	12	CENPF, CHEK1, CHEK2, FANCD2, GINS2, MCM6, MSH2, RAD54L, RFC4, RRM2, SMC2, TOP2A	C
PETROVA_PROX1_TARGETS_UP	C/N_up & C/P_up	5	BUB1B, CCNE2, CDK1, MCM6, TOP2A	C & C/P
BROWNE_HCMV_INFECTION_2HR_UP	P/N_up	4	EGR1, FOS, NR4A2, NR4A3	P/N
ENK_UV_RESPONSE_EPIDERMIS_DN	P/N_up	10	AREG, CH25H, EGR1, EGR2, FJX1, GSN, LXN, NR4A2, PTGS2, STK17B	P/N
GSE9988_LOW_LPS_VS_CTRL_TREATED_MONOCYTE_UP	P/N_up	8	AREG, EGR1, IL6, NR4A2, NR4A3, PTGS2, RGS1, SERPINB8	P/N
MODULE_43	C/N_down	17	ACADL, ACADSB, CAT, CYP1A1, CYP26A1, CYP2C8, CYP3A4, CYP3A7, CYP4A11, DAO, ETFDH, FMO3, GYS2, LEPR, PPP1R1A, QDPR, TREH	C
MODULE_99	C/N_down	17	ABAT, AFM, BBOX1, BCHE, CRHBP, F11, FETUB, IGF1, LEPR, LIFR, MARCO, NAT2, OTC, PCK1, SLC10A1, SLC22A1, UGT8	C/N
PKCA_DN.V1_UP	C/N_down	17	ADRA1A, BBOX1, CYP2C18, CYP2C8, DIO1, FBP1, LECT2, NR1I3, PCK1, PIPOX, PRODH2, RDH16, RDH5, SEC14L2, SLC2A2, TREH, UPB1	C

*"Profile": the expression profile used for prognosis; "C" means the absolute expression value of genes in cancer; while "C/P" and "P/N" mean the relative gene expression level of cancer/pericancerous liver and pericancerous/normal liver respectively.

The first three gene sets in Table 1 showed prognosis potential with up-regulated DEGs in cancer liver. Their DEG members expression level in cancer could be used for prognosis in both our 45 HCC patients from [GEO:GSE45114] and the 227 HCC patients from [GEO:GSE14520] (P < 0.05 and HR > 0). The positive HR (hazard ratio) means the higher DEGs expression the worse the prognosis. In Figure 2, we show the prognosis ability of nine DEGs in gene set "chr1q32" which was reported to be the most recurrently gained genomic region in HCC [60]. Another gene set "KAUFFMANN_MELANOMA_RELAPSE_UP" [61] contains DNA repair and replication related genes (Additional file 6).

The next three gene sets in Table 1 showed prognosis potential of pericancerous liver with up-regulated DEGs. Gene set "BROWNE_HCMV_INFECTION_2HR_UP" contains genes that were related to hepatic inflammation and cirrhosis [62]. Their expression level may represent not only inflammation and cirrhosis but also carcinogenesis of HCC (Figure 3). And the gene set "ENK_UV_RESPONSE_EPIDERMIS_DN" [63] contains genes related to DNA damage repair (Additional file 6).

Besides up-regulated DEGs, the down-regulated DEGs in cancer liver also showed prognosis potential in the last three gene sets (Table 1 and Additional file 6). Here, negative HR (hazard ratio) means the lower DEG expression the worse the prognosis.

In summary, prognosis of HCC patients could be predicted with expression profiles of both up-regulated DEGs and down-regulated DEGs enriched in certain functional gene sets.

### Association between microRNA expression profile and postoperative survival time

Gene sets enriched with DEGs either in C/N, C/P or P/N were shown to have prognosis potential, as reported above. MicroRNA profiling data is also available for the 45 HCC patients with paired pericancer/cancer samples. Since microRNA expression signatures in hepatocellular carcinoma have been stated to possess prognostic value before [17, 64], we would like to see in our work, whether DEGs related microRNA sets could be prognostic. We identified the targeting relationships between microRNAs and genes that appear in at least three microRNA target databases from six: miRBase [35], TargetScan [36], miRanda [37], tarBase [38], mirTarget2 [39] and PicTar [40]. The microRNAs that target DEGs in each enriched gene set comprise a candidate microRNA set for prognosis prediction. Then we tested the association between the expression of these microRNAs and postoperative survival times in our 45 patients from [GEO:GSE10694]. Two prognostic microRNA gene sets were validated in an independent test dataset TCGA LIHC (27 HCC patients with RNASeq data) (Table 2).

**Table 2:** Gene sets associated to postoperative survival time with microRNA expression profile (validated in TCGA LIHC).

Gene set *	DEG	microRNAs targeting to DEG	Count
SMID_BREAST_CANCER_BASAL_DN	ABAT ASPA CXCL14 ESR1 FAM134B IGF1 PBLD PCK1 SORD	hsa-let-7b, hsa-miR-130a, hsa-miR-130b, hsa-miR-136, hsa-miR-148a, hsa-miR-148b, hsa-miR-152, hsa-miR-181c, hsa-miR-181d, hsa-miR-182, hsa-miR-18a, hsa-miR-18b, hsa-miR-19a, hsa-miR-206, hsa-miR-22, hsa-miR-221, hsa-miR-222, hsa-miR-26b, hsa-miR-29a, hsa-miR-29c, hsa-miR-302a, hsa-miR-302b, hsa-miR-302c, hsa-miR-302d, hsa-miR-31, hsa-miR-340, hsa-miR-372, hsa-miR-373, hsa-miR-410, hsa-miR-425, hsa-miR-488, hsa-miR-495, hsa-miR-506, hsa-miR-520b, hsa-miR-520e, hsa-miR-93, hsa-miR-96	9 Genes 37 miR
SMID_BREAST_CANCER_LUMINAL_B_UP	ABAT ESR1 FAM134B SORD	hsa-miR-130a, hsa-miR-130b, hsa-miR-148a, hsa-miR-148b, hsa-miR-152, hsa-miR-181c, hsa-miR-181d, hsa-miR-182, hsa-miR-18a, hsa-miR-18b, hsa-miR-19a, hsa-miR-22, hsa-miR-221, hsa-miR-222, hsa-miR-302a, hsa-miR-302c, hsa-miR-302d, hsa-miR-31, hsa-miR-372, hsa-miR-506, hsa-miR-93, hsa-miR-96	4 Genes 22 miRs

*These two gene sets were both enriched with C/N_down group DEGs. And the absolute expression value of microRNA in cancer, which detected by array in [GEO:GSE10694] or by RNASeq in TCGA LIHC were used for prognosis.

**Figure 4:**
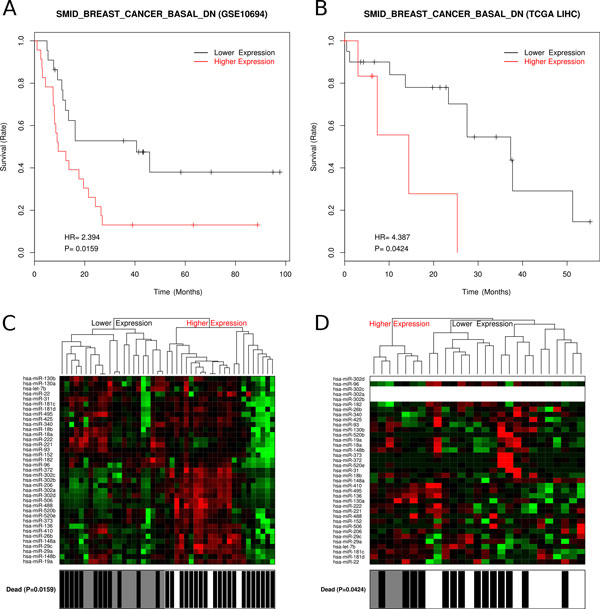
**Prognosis with DEG-members-targeting-microRNAs in gene set "SMID_BREAST_CANCER_BASAL_DN"**. Gene set "SMID_BREAST_CANCER_BASAL_DN" could be used for prognosis with DEG-members-targeting-microRNA expression levels in HCC. Kaplan-Meier survival curves and heatmaps of the correlation between the postoperative survival time and the expression profile of microRNAs that target differentially expressed gene members in the gene set "SMID_BREAST_CANCER_BASAL_DN". A. Kaplan-Meier survival curve of DEG-targeting-microRNA expression levels in 45 HCC patients from [GEO:GSE10694]. B. Kaplan-Meier survival curve of DEG-targeting-microRNA expression levels in 27 HCC patients from TCGA LIHC. C. Heatmap of DEG-targeting-microRNA expression levels from [GEO:GSE10694]. D. Heatmap of DEG-targeting-microRNA expression levels from TCGA LIHC.

Gene set "SMID_BREAST_CANCER_BASAL_DN" contains genes that are down-regulated in basal subtype of breast cancer samples [65]. We found that 32 member genes were down-regulated in HCC relative to normal liver and nine of them were targeted by 37 microRNAs. The 37 microRNAs expression profile in cancer liver could be used for prognosis (Figure 4). The positive HR (hazard ratio) means the higher expression the worse prognosis.

The other gene set "SMID_BREAST_CANCER_LUMINAL_B_UP" contains genes that are up-regulated in the luminal B subtype of breast cancer [65]. Its 12 member genes were down-regulated in HCC relative to normal liver and four of them were targeted by 22 microRNAs. The 22 microRNAs expression profile in the cancer liver could be used for prognosis (Additional file 6). Interestingly, the four DEGs are subset of the nine DEGs mentioned in above gene set (Table 2), which shows the similarity and difference between basal subtype and luminal subtype of breast cancers.

Most of the microRNAs listed in Table 2 have been annotated to be related to HCC in The human microRNA disease database (HMDD) [66] (Additional file 7). Such as the cell proliferation related microRNAs: hsa-mir-18a, hsa-mir-93, hsa-mir-96; and cancer recurrence related microRNAs: hsa-mir-148a, hsa-mir-18a, hsa-mir-18b, hsa-mir-19a, hsa-mir-22, hsa-mir-221, hsa-mir-222, hsa-mir-96. In Table 2 there are some microRNAs have not been recorded to be HCC related by HMDD, including: hsa-miR-136, hsa-miR-206, hsa-miR-26b, hsa-miR-302a, hsa-miR-302d, hsa-miR-340, hsa-miR-410, hsa-miR-488, hsa-miR-495, hsa-miR-506. They may be potentially HCC related.

## Discussion

There have been numerous studies of hepatocellular carcinoma(HCC) in comparison with pericancerous tissue as normal control, in the purpose of identifying differentially expressed genes, modules, networks etc., in order to find cancer biomarkers, cluster samples, or to predict prognosis. Such studies especially on Chinese HCC patient samples take on a strong assumption that pericancerous liver tissue of HCC is normal, while this in a large percentage is wrong. Most patients diagnosed with HCC in China already went through years of liver cirrhotic change because of chronic HBV infection, alcoholism, or fatty liver etc. Therefore, in this work of ours, we designed a set of normal liver tissues as control. With such a design, we were able to identify differentially expressed genes (DEGs) with a gradual up-regulation from normal to pericancerous to cancerous liver, or DEGs with a gradual down-regulation. Further gene set enrichment analysis (GSEA) on GO terms, pathway, and transcription factor targets suggested the main up-regulated trend to be in cell cycle and proliferation, and the main down-regulated trend to be metabolism. Although such conclusions may not be totally novel, it is nice to see such direct proof of gradual molecular transitions in liver carcinogenesis. More in-depth detailed analyses of the gradually changed gene sets may even lead to clues for early diagnosis, however it is beyond the scope of this paper's discussion.

Instead, we made efforts to testify whether gene sets enriched with gradually changing DEGs have prognostic value. Many previous researches proposed lists of DEGs, pathways, network modules (the latter two can be considered as gene sets) to predict prognosis for HCC patients. We used somewhat a combined approach. Instead of using groups of single DEGs that would lack functional binding, or full gene sets that would contain too many genes, we used DEGs grouped in preselected enriched gene sets as classifiers. The advantage is that the classifier is relatively small, and the DEGs share a common gene function family. Indeed we were able to identify nine such gene set DEGs classifiers possessing prognostic prediction power, and could even be validated in an independent dataset with larger patient number. Quite a few such gene sets behold cell proliferation or DNA repair functions in liver cancer tissues, or inflammation function in pericancerous liver tissues.

MicroRNA (miRNA) as a new kind of regulatory biomarker, has been investigated in many cancers in recent years. In our previous works, individual miRNAs and miRNA regulatory network modules have been successfully applied in HCC prognosis prediction [17, 67, 68]. In this work, we took a simple approach. Since some of the gene sets enriched with gradually changing DEGs in liver carcinogenesis have been proved to possess prognosis potential, we substituted such gene sets with miRNAs targeting the DEGs they contained. To ensure the substitutions are relevant all miRNA-DEG target relationships must be carefully curated from multiple databases and prediction algorithms. Two gene sets substituted with miRNAs acquired prognostic power, and could be validated in a TCGA RNASeq dataset which has miRNA expression data of paired HCC samples available. This may actually represent a simple approach to quickly discover relevant miRNAs which might have caused the dysregulation of the DEGs that are associated with prognosis. Traditionally differentially expressed miRNAs should first be detected and secondly correlated to their downstream targets and further to functional applications.

Figure 1 and Additional file 3 indicated the similarities between pericancerous and normal liver, when comparing to HCC. This proves the rationality for many researchers who take pericancerous tissues as control. Similarly, researchers found that gene expression pattern is more significantly related to physiological condition rather than tissue spatial distance [69]. They reported that different cancer tissues may show common gene expression patterns. Our results might provide an evidence for that: some prognosis biomarkers we found in HCC also play important roles in other cancers, such as melanoma and breast cancer (Additional file 6 and Figure 4). At the same time, we found that pericancerous liver shared some characters of HCC, which provided the probability for prognosis prediction with gene expression profiles of pericancerous liver (Figure 3 and Additional file 6).

There are of course limitations to our work. The patient sample size is not big, and the normal samples are from healthy individuals who died accidentally, rather than real normal liver sample of the same HCC patient, which is hardly possible to get. Therefore the gradual changes from normal to pericancerous to cancerous liver tissues observed in this dataset may not be very steady accessible features that can be easily applied clinically. However our strategy does put an emphasis on the importance to study the cirrhotic and inflammatic nature of pericancerous tissue in HCC patients, which show both carcinogenesis trend and prognostic potential. In the future, integrating sequence information from DNASeq and RNASeq as well as clinical information in bigger sample size data sets may benefit such purpose.

## Conclusions

In this work, Based on differentially expressed genes (DEGs) detected from normal, pericancerous, cancerous liver samples by array technology, and the annotated gene sets from GSEA MSigDB, we managed to show some molecular transitional changes represented by different GO, pathway, regulatory gene sets. DEGs profile of nine of such gene sets could be applied to predict hepatocellular carcinoma (HCC) patient survival. Two gene sets acquired prognostic capacity after being substituted with microRNAs targeting the DEGs contained in the original gene set. Both gene set prognosis and miRNA set prognosis were validated with independent HCC patients gene expression or RNASeq dataset. Our work represents an effort to study pericancerous nature of HCC, and a simple way to identify relevant regulatory miRNAs to DEGs.

## Electronic supplementary material

Additional file 1: **Tables, phenotypic data of HCC patients**. Phenotypic data of: 45 patients from [GEO:GSE45114] and [GEO:GSE10694]; 227 patients from [GEO:GSE14520]; 27 patients from TCGA LIHC. (XLS 76 KB)

Additional file 2: **Tables, DEGs and gene sets enriched with DEGs**. The 551 identified differentially expressed genes (DEGs) of three comparisons: Cancer/Normal (C/N), Cancer/Pericancerous (C/P) and Pericancerous/Normal (P/N). The value "NA" means that this gene (row head) is not a DEG in this comparison (column head). And the 868 non repetitive gene sets that enriched with the nine groups of DEGs (nine circles in Figure 1) by both two enrich method (hypergeometric test and GSEA). The value "NA" means that this gene set (row head) is not enriched with this group of DEGs (column head). (XLS 396 KB)

Additional file 3: **Figure, venn diagram of gene sets enriched with DEGs from three comparisons**. Venn diagram of gene sets enriched with DEGs from three comparisons: Cancer/Normal (C/N), Pericancerous/Normal (P/N) and Cancer/Pericancerous (C/P). A. Venn diagram of gene sets enriched with the all DEGs from three comparisons. B. Venn diagram of gene sets enriched with the up-regulated DEGs from three comparisons. The red number showed the number of gene sets enriched with both C/N_up DEGs and C/P_up DEGs. C. Venn diagram of gene sets enriched with the down-regulated DEGs from three comparisons. The blue number showed the number of gene sets enriched with both C/N_down DEGs and C/P_down DEGs. D. Counts of gene ontology, pathway and transcription factor targets gene sets enriched with both C/N DEGs and C/P DEGs. The numbers in red were covered by red number in subgraph B. The numbers in blue were covered by blue number in subgraph C. (PDF 12 KB)

Additional file 4: **Figures, venn diagram of GOs, Pathways and TFTs enriched with DEGs from three comparisons**. Venn diagram of gene sets about gene ontology terms (GO), Pathways and Transcription factor targets (TFT) enriched with DEGs from three comparisons: Cancer/Normal (C/N), Pericancerous/Normal (P/N) and Cancer/Pericancerous (C/P). A. Venn diagram of gene sets enriched with the all DEGs from three comparisons. B. Venn diagram of gene sets enriched with the up-regulated DEGs from three comparisons. The red number showed the number of gene sets enriched with both C/N_up DEGs and C/P_up DEGs. C. Venn diagram of gene sets enriched with the down-regulated DEGs from three comparisons. The blue number showed the number of gene sets enriched with both C/N_down DEGs and C/P_down DEGs. (PDF 41 KB)

Additional file 5: **Tables, gene sets enriched with both C/N DEGs and C/P DEGs**. Gene sets about Gene ontology terms (GOs), Pathways and Transcription factor targets gene sets (TFTs) enriched with both C/N DEGs and C/P DEGs. (XLS 60 KB)

Additional file 6: **Figures, gene sets used for prognosis with expression profile of DEG members**. Kaplan-Meier survival curves and heatmaps of the correlation between the postoperative survival time and the expression profile of DEG members in the gene set "KAUFFMANN_MELANOMA_RELAPSE_UP", "PETROVA_PROX1_TARGETS_UP", "ENK_UV_RESPONSE_EPIDERMIS_DN", "GSE9988_LOW_LPS_VS_CTRL_TREATED_MONOCYTE_UP", "MODULE_43", "MODULE_99" and "PKCA_DN.V1_UP". Each figure includs four subgraphs: A. Kaplan-Meier survival curve of DEG expression levels in 45 HCC patients from [GEO:GSE45114]. B. Kaplan-Meier survival curve of DEG expression levels in 227 HCC patients from [GEO:GSE14520]. C. Heatmap of DEG expression levels in 45 HCC patients from [GEO:GSE45114]. D. Heatmap of DEG expression levels in 227 HCC patients from [GEO:GSE14520]. And the Kaplan-Meier survival curves and heatmaps of the correlation between the postoperative survival time and expression profile of DEG-targeting microRNAs in gene set "SMID_BREAST_CANCER_LUMINAL_B_UP" which validated with 27 HCC patients from TCGA LIHC. Including four subgraphs: A. Kaplan-Meier survival curve of DEG expression levels in 45 HCC patients from [GEO:GSE10694]. B. Kaplan-Meier survival curve of DEG expression levels in 27 HCC patients from TCGA LIHC. C. Heatmap of DEG expression levels in 45 HCC patients from [GEO:GSE10694]. D. Heatmap of DEG expression levels in 27 HCC patients from TCGA LIHC. (Note: The positive HR (hazard ratio) means the higher expression the worse prognosis. While the negative HR (hazard ratio) means the lower expression the worse prognosis. Some genes may not appear in subgraph D, because those genes (or microRNAs) were not detected in [GEO:GSE14520] (or TCGA LIHC). The remaining DEGs (or microRNAs) still show significant potential for prognosis.) (PDF 870 KB)

Additional file 7: **Table, microRNAs annotation recorded by the human microRNA disease database (HMDD)**. Most microRNAs listed in Table 2 have been annotated by the human microRNA disease database (HMDD). The table list the 27 HCC related microRNAs with their references and descriptions annotated by HMDD. (XLS 165 KB)

## List of abbreviations used

HCC: Hepatocellular carcinoma
DEG: Differentially expressed genes
GSEA: Gene set enrichment analysis
GEO: Gene expression omnibus
MSigDB: Molecular signatures database
GO: Gene ontology
TCGA: The cancer genome atlas
LIHC: Liver hepatocellular carcinoma
C/N: Cancer/Normal
C/P: Cancer/Pericancerous
P/N: Pericancerous/Normal
TFT: Transcription factor targets
HR: Hazard ratio
HMDD: Human microRNA disease database.
