# Supplementary material for: Application of microRNA and mRNA expression profiling on prognostic biomarker discovery for hepatocellular carcinoma
Source: BMC Genomics. 2014 Jan 24;15(Suppl 1):S13. doi: 10.1186/1471-2164-15-S1-S13 (PMC4046763; doi:10.1186/1471-2164-15-S1-S13)
Supplement: Supplementary file 3 — Additional file 3: Figure, venn diagram of gene sets enriched with DEGs from three comparisons. Venn diagram of gene sets enriched with DEGs from three comparisons: Cancer/Normal (C/N), Pericancerous/Normal (P/N) and Cancer/Pericancerous (C/P). A. Venn diagram of gene sets enriched with the all DEGs from three comparisons. B. Venn diagram of gene sets enriched with the up-regulated DEGs from three comparisons. The red number showed the number of gene sets enriched with both C/N_up DEGs and C/P_up DEGs. C. Venn diagram of gene sets enriched with the down-regulated DEGs from three comparisons. The blue number showed the number of gene sets enriched with both C/N_down DEGs and C/P_down DEGs. D. Counts of gene ontology, pathway and transcription factor targets gene sets enriched with both C/N DEGs and C/P DEGs. The numbers in red were covered by red number in subgraph B. The numbers in blue were covered by blue number in subgraph C. (PDF 12 KB) [file 12864_2014_5686_MOESM3_ESM.pdf]

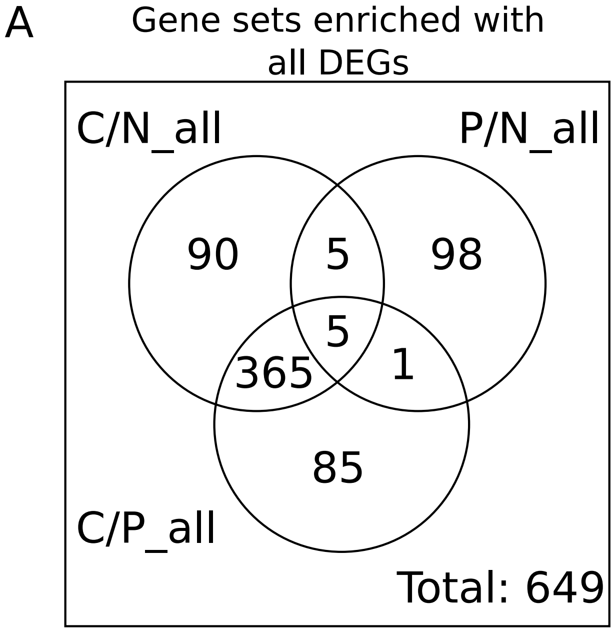

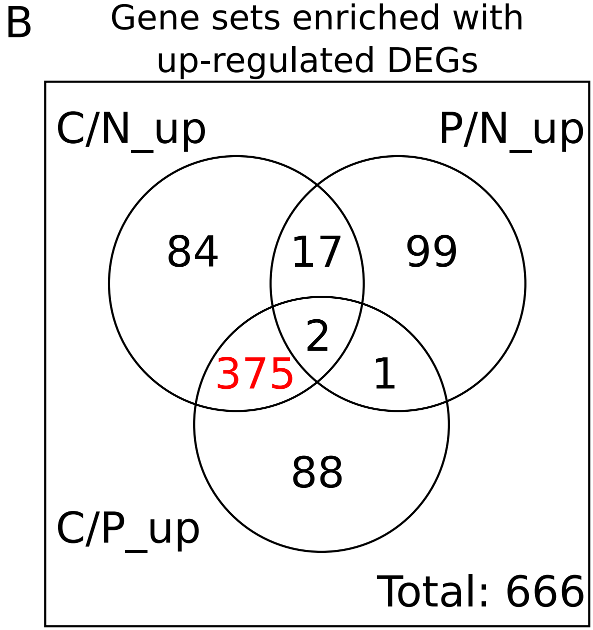

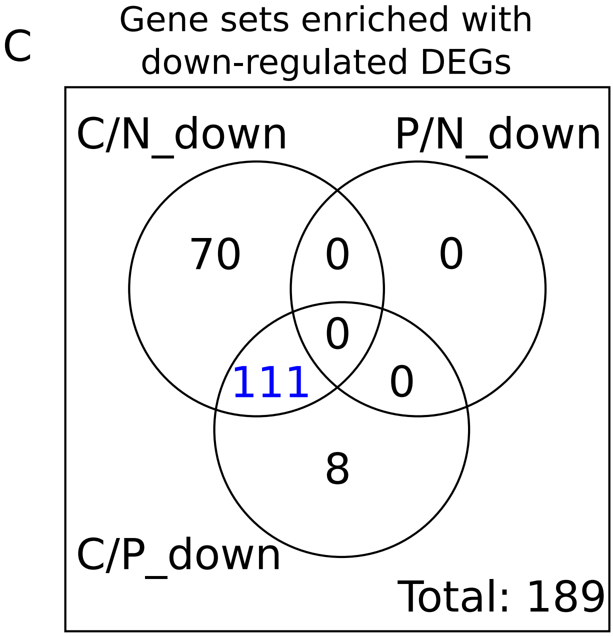

D

Gene sets enriched with both C/N DEGs and C/P DEGs

Gene Set	C/N and C/P	
	Up	Down
Gene Ontology	19	21
Pathway	24	21
Transcription Factor Targets	19	1
