# Supplementary material for: Application of microRNA and mRNA expression profiling on prognostic biomarker discovery for hepatocellular carcinoma
Source: BMC Genomics. 2014 Jan 24;15(Suppl 1):S13. doi: 10.1186/1471-2164-15-S1-S13 (PMC4046763; doi:10.1186/1471-2164-15-S1-S13)
Supplement: Supplementary file 4 — Additional file 4: Figures, venn diagram of GOs, Pathways and TFTs enriched with DEGs from three comparisons. Venn diagram of gene sets about gene ontology terms (GO), Pathways and Transcription factor targets (TFT) enriched with DEGs from three comparisons: Cancer/Normal (C/N), Pericancerous/Normal (P/N) and Cancer/Pericancerous (C/P). A. Venn diagram of gene sets enriched with the all DEGs from three comparisons. B. Venn diagram of gene sets enriched with the up-regulated DEGs from three comparisons. The red number showed the number of gene sets enriched with both C/N_up DEGs and C/P_up DEGs. C. Venn diagram of gene sets enriched with the down-regulated DEGs from three comparisons. The blue number showed the number of gene sets enriched with both C/N_down DEGs and C/P_down DEGs. (PDF 41 KB) [file 12864_2014_5686_MOESM4_ESM.pdf]

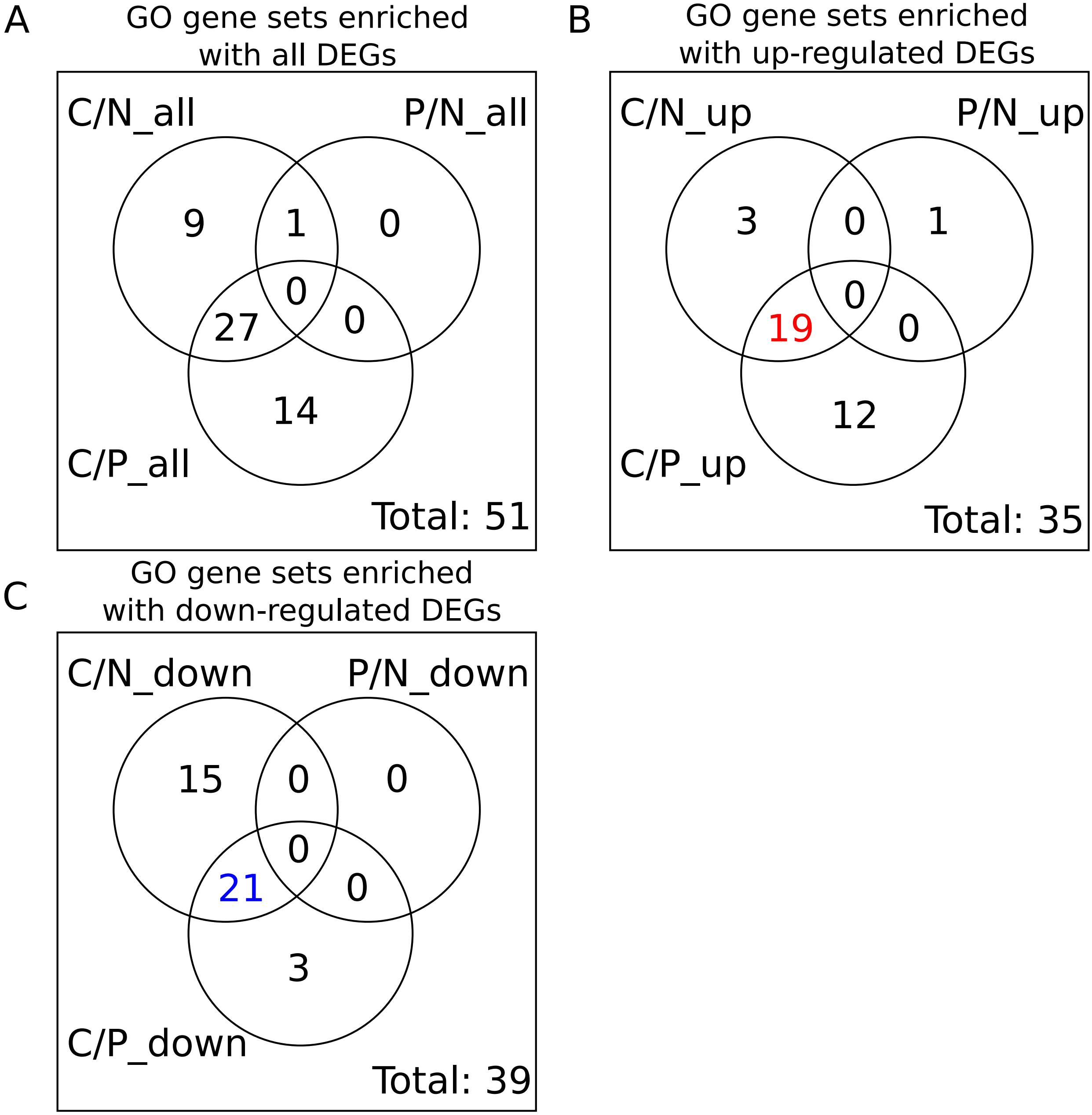
A
GO gene sets enriched
with all DEGs
C/N_all
P/N_all
9
1
0
0
27
0
14
C/P_all
Total: 51
B
GO gene sets enriched
with up-regulated DEGs
C/N_up
P/N_up
3
0
1
0
19
0
12
C/P_up
Total: 35
C
GO gene sets enriched
with down-regulated DEGs
C/N_down
P/N_down
15
0
0
0
21
0
3
C/P_down
Total: 39

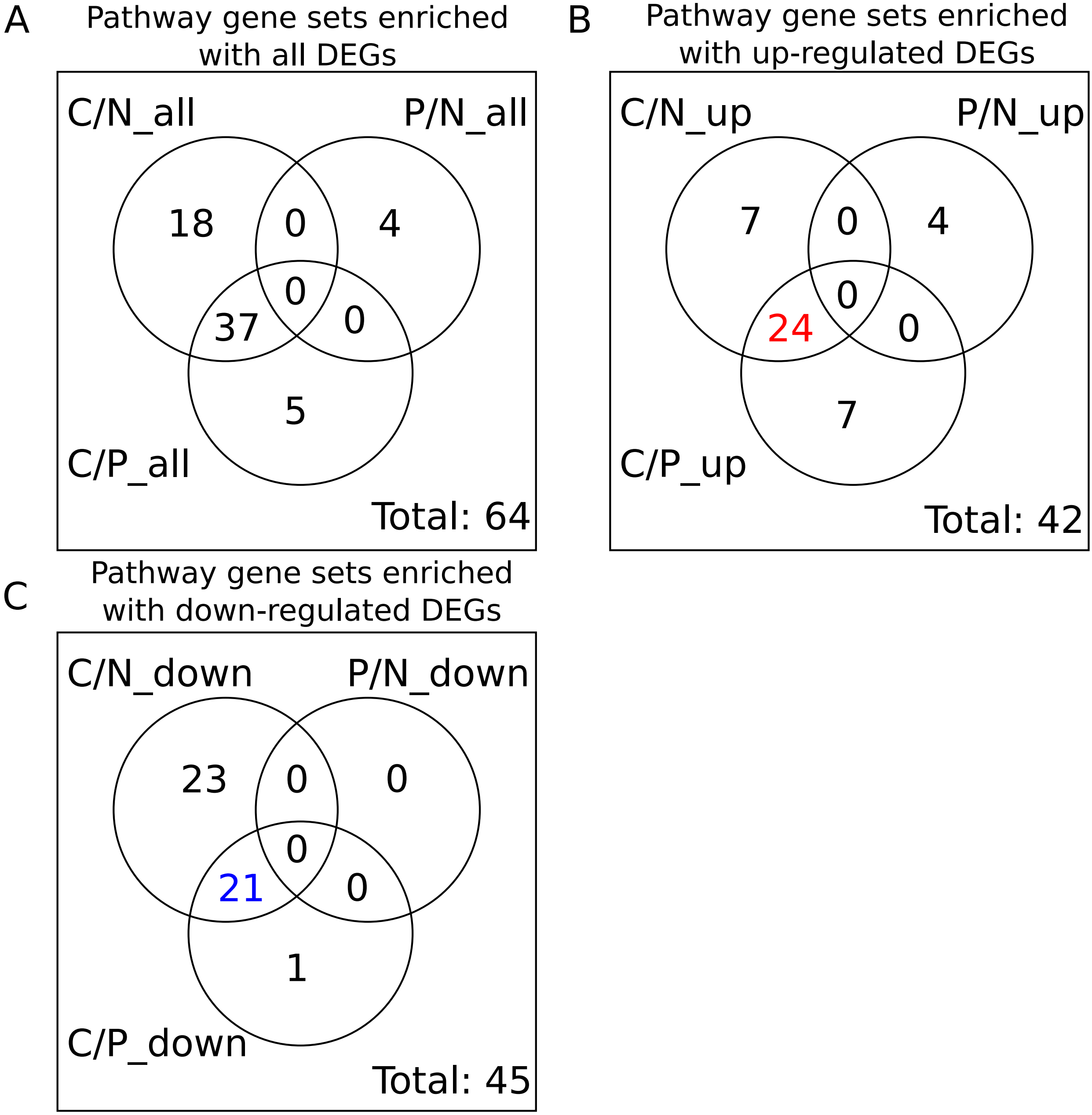

A
Pathway gene sets enriched with all DEGs
C/N_all
P/N_all
18
0
4
0
37
0
5
C/P_all
Total: 64
B
Pathway gene sets enriched with up-regulated DEGs
C/N_up
P/N_up
7
0
4
0
24
0
7
C/P_up
Total: 42
C
Pathway gene sets enriched with down-regulated DEGs
C/N_down
P/N_down
23
0
0
0
21
0
1
C/P_down
Total: 45

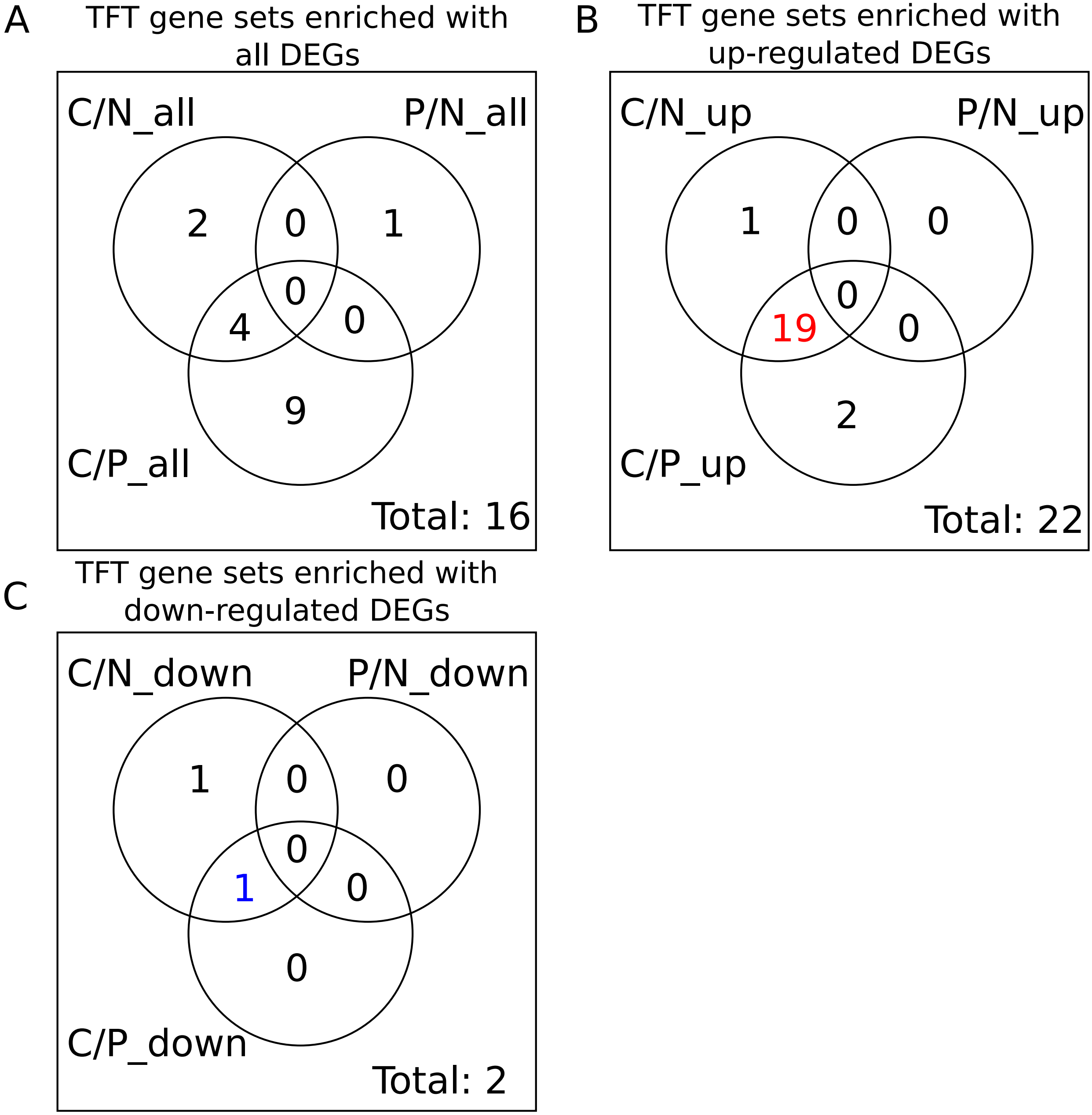
A
TFT gene sets enriched with all DEGs
C/N_all
P/N_all
C/P_all
2
0
1
0
4
0
9
Total: 16
B
TFT gene sets enriched with up-regulated DEGs
C/N_up
P/N_up
C/P_up
1
0
0
0
19
0
2
Total: 22
C
TFT gene sets enriched with down-regulated DEGs
C/N_down
P/N_down
C/P_down
1
0
0
0
1
0
0
Total: 2
