# Supplementary material for: Application of microRNA and mRNA expression profiling on prognostic biomarker discovery for hepatocellular carcinoma
Source: BMC Genomics. 2014 Jan 24;15(Suppl 1):S13. doi: 10.1186/1471-2164-15-S1-S13 (PMC4046763; doi:10.1186/1471-2164-15-S1-S13)
Supplement: Supplementary file 6 — Additional file 6: Figures, gene sets used for prognosis with expression profile of DEG members. Kaplan-Meier survival curves and heatmaps of the correlation between the postoperative survival time and the expression profile of DEG members in the gene set "KAUFFMANN_MELANOMA_RELAPSE_UP", "PETROVA_PROX1_TARGETS_UP", "ENK_UV_RESPONSE_EPIDERMIS_DN", "GSE9988_LOW_LPS_VS_CTRL_TREATED_MONOCYTE_UP", "MODULE_43", "MODULE_99" and "PKCA_DN.V1_UP". Each figure includs four subgraphs: A. Kaplan-Meier survival curve of DEG expression levels in 45 HCC patients from [GEO:GSE45114]. B. Kaplan-Meier survival curve of DEG expression levels in 227 HCC patients from [GEO:GSE14520]. C. Heatmap of DEG expression levels in 45 HCC patients from [GEO:GSE45114]. D. Heatmap of DEG expression levels in 227 HCC patients from [GEO:GSE14520]. And the Kaplan-Meier survival curves and heatmaps of the correlation between the postoperative survival time and expression profile of DEG-targeting microRNAs in gene set "SMID_BREAST_CANCER_LUMINAL_B_UP" which validated with 27 HCC patients from TCGA LIHC. Including four subgraphs: A. Kaplan-Meier survival curve of DEG expression levels in 45 HCC patients from [GEO:GSE10694]. B. Kaplan-Meier survival curve of DEG expression levels in 27 HCC patients from TCGA LIHC. C. Heatmap of DEG expression levels in 45 HCC patients from [GEO:GSE10694]. D. Heatmap of DEG expression levels in 27 HCC patients from TCGA LIHC. (Note: The positive HR (hazard ratio) means the higher expression the worse prognosis. While the negative HR (hazard ratio) means the lower expression the worse prognosis. Some genes may not appear in subgraph D, because those genes (or microRNAs) were not detected in [GEO:GSE14520] (or TCGA LIHC). The remaining DEGs (or microRNAs) still show significant potential for prognosis.) (PDF 870 KB) [file 12864_2014_5686_MOESM6_ESM.pdf]

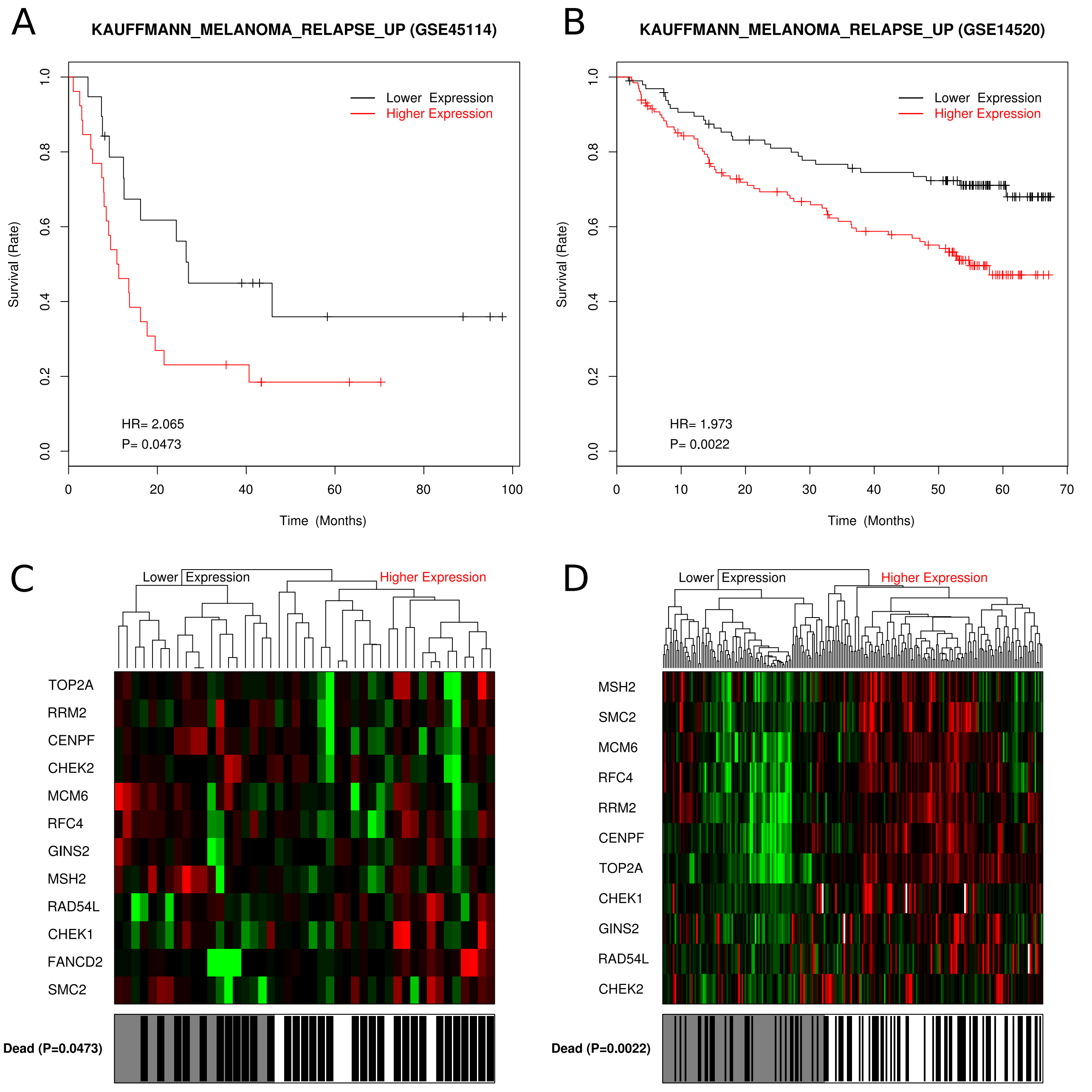

A
KAUFFMANN_MELANOMA_RELAPSE_UP (GSE45114)
Lower Expression
Higher Expression
HR= 2.065
P= 0.0473
Survival (Rate)
Time (Months)
B
KAUFFMANN_MELANOMA_RELAPSE_UP (GSE14520)
Lower Expression
Higher Expression
HR= 1.973
P= 0.0022
Survival (Rate)
Time (Months)
C
Lower Expression
Higher Expression
TOP2A
RRM2
CENPF
CHEK2
MCM6
RFC4
GINS2
MSH2
RAD54L
CHEK1
FANCD2
SMC2
Dead (P=0.0473)
D
Lower Expression
Higher Expression
MSH2
SMC2
MCM6
RFC4
RRM2
CENPF
TOP2A
CHEK1
GINS2
RAD54L
CHEK2
Dead (P=0.0022)

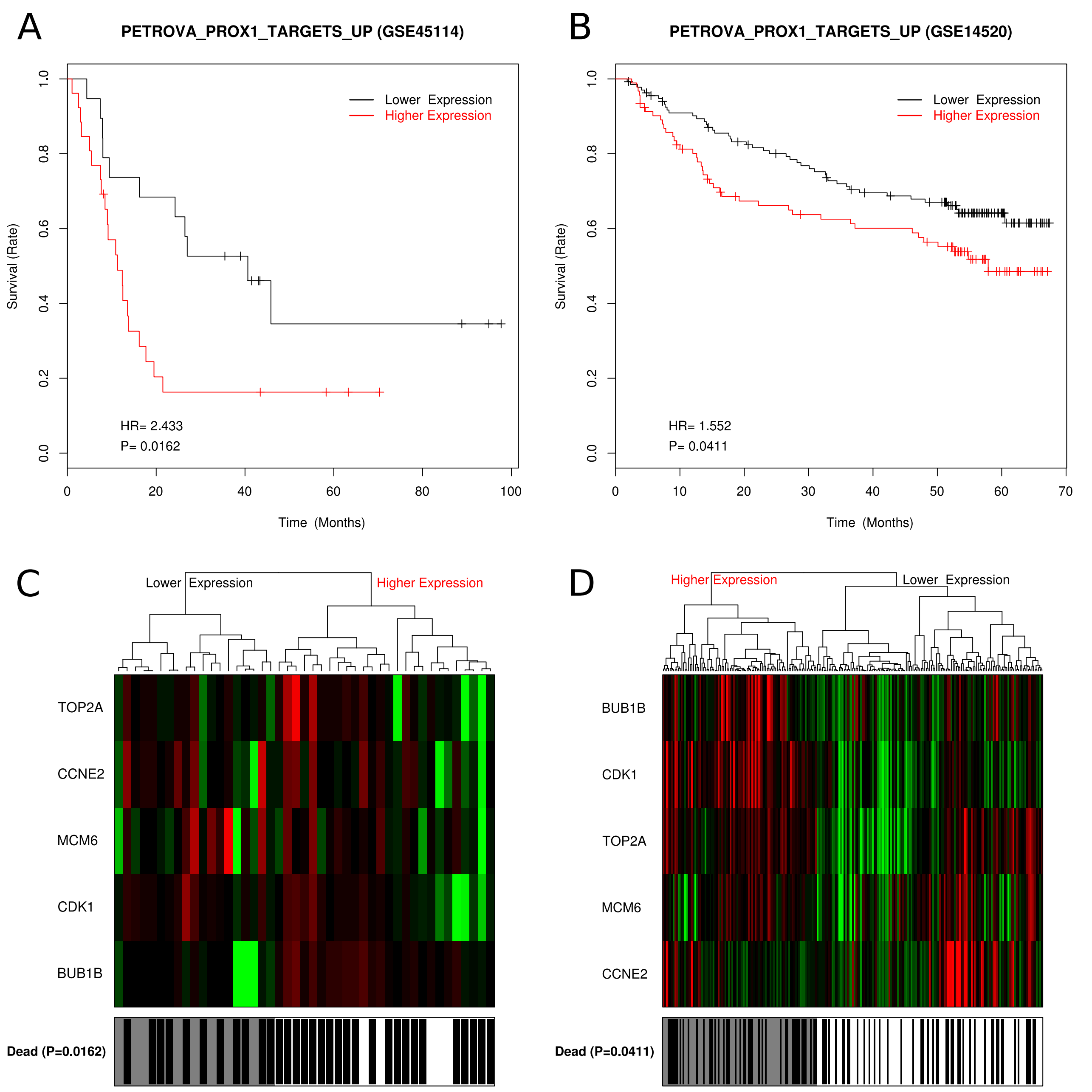

A
PETROVA_PROX1_TARGETS_UP (GSE45114)
Lower Expression
Higher Expression
HR= 2.433
P= 0.0162
Survival (Rate)
Time (Months)
B
PETROVA_PROX1_TARGETS_UP (GSE14520)
Lower Expression
Higher Expression
HR= 1.552
P= 0.0411
Survival (Rate)
Time (Months)
C
Lower Expression
Higher Expression
TOP2A
CCNE2
MCM6
CDK1
BUB1B
Dead (P=0.0162)
D
Higher Expression
Lower Expression
BUB1B
CDK1
TOP2A
MCM6
CCNE2
Dead (P=0.0411)

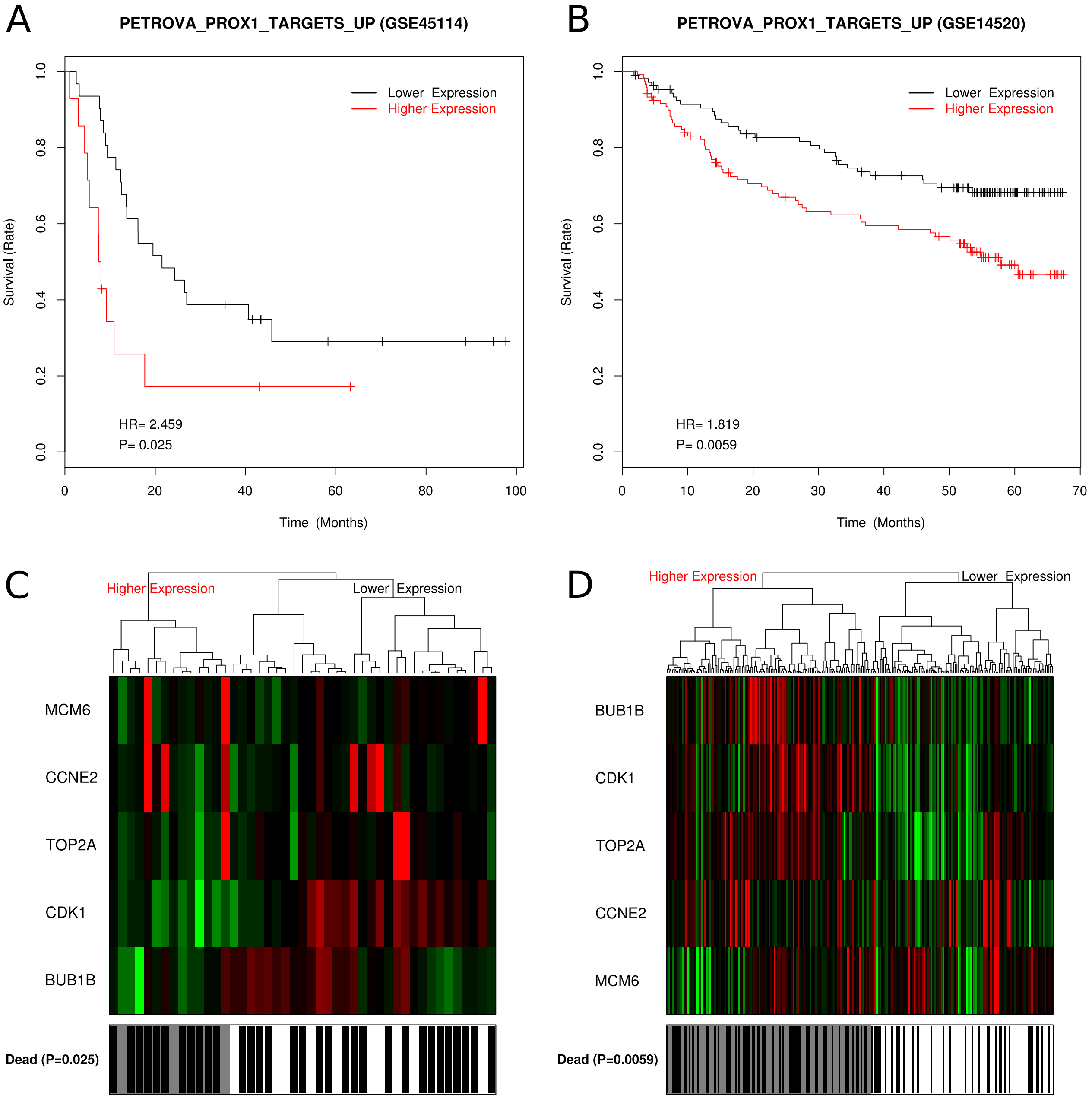

A
PETROVA_PROX1_TARGETS_UP (GSE45114)
Lower Expression
Higher Expression
HR= 2.459
P= 0.025
Survival (Rate)
Time (Months)
B
PETROVA_PROX1_TARGETS_UP (GSE14520)
Lower Expression
Higher Expression
HR= 1.819
P= 0.0059
Survival (Rate)
Time (Months)
C
Higher Expression
Lower Expression
MCM6
CCNE2
TOP2A
CDK1
BUB1B
Dead (P=0.025)
D
Higher Expression
Lower Expression
BUB1B
CDK1
TOP2A
CCNE2
MCM6
Dead (P=0.0059)

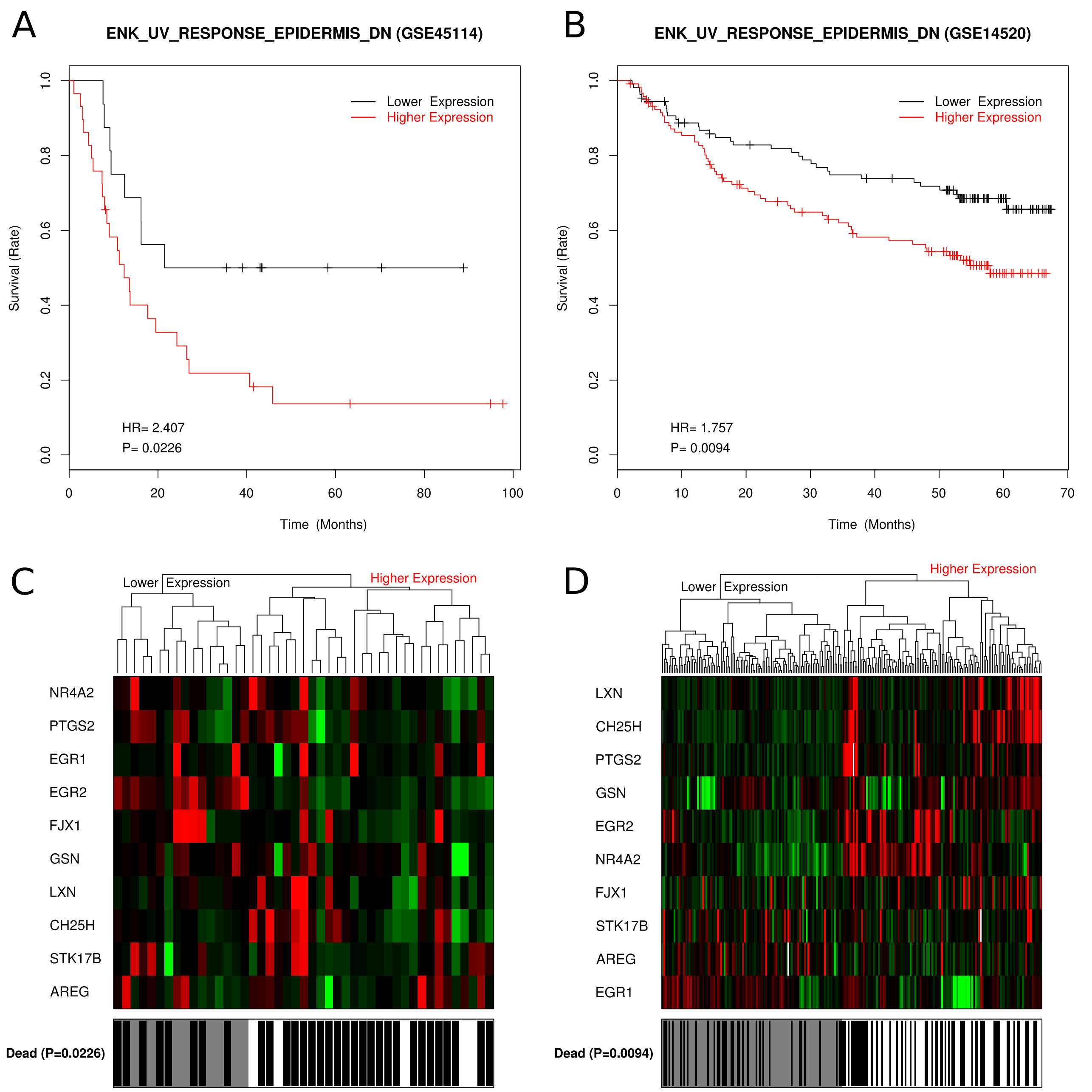

A
ENK_UV_RESPONSE_EPIDERMIS_DN (GSE45114)
Survival (Rate)
Time (Months)
Lower Expression
Higher Expression
HR= 2.407
P= 0.0226
B
ENK_UV_RESPONSE_EPIDERMIS_DN (GSE14520)
Survival (Rate)
Time (Months)
Lower Expression
Higher Expression
HR= 1.757
P= 0.0094
C
Lower Expression
Higher Expression
NR4A2
PTGS2
EGR1
EGR2
FJX1
GSN
LXN
CH25H
STK17B
AREG
Dead (P=0.0226)
D
Lower Expression
Higher Expression
LXN
CH25H
PTGS2
GSN
EGR2
NR4A2
FJX1
STK17B
AREG
EGR1
Dead (P=0.0094)

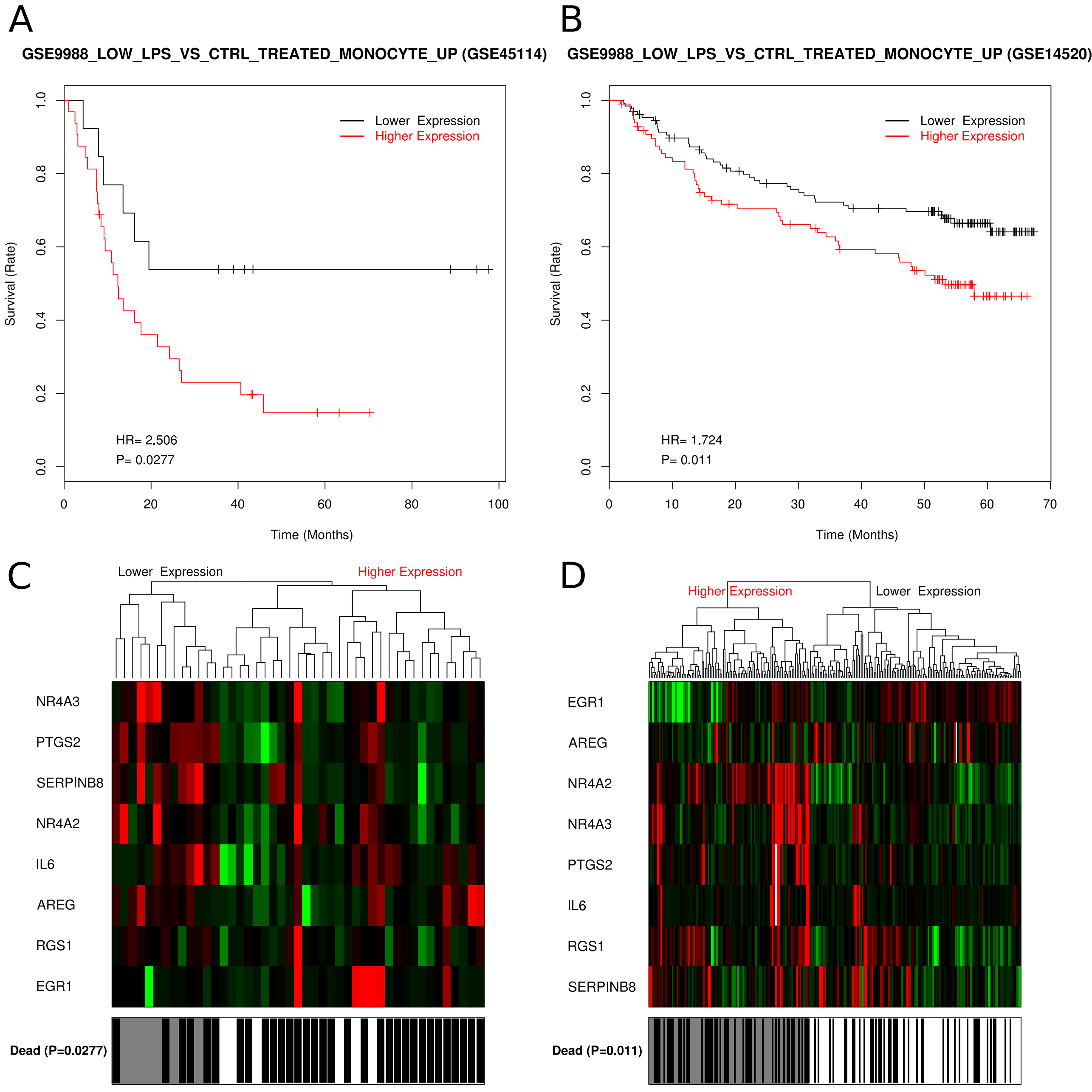

A
GSE9988_LOW_LPS_VS_CTRL_TREATED_MONOCYTE_UP (GSE45114)
Lower Expression
Higher Expression
HR= 2.506
P= 0.0277
Survival (Rate)
Time (Months)
B
GSE9988_LOW_LPS_VS_CTRL_TREATED_MONOCYTE_UP (GSE14520)
Lower Expression
Higher Expression
HR= 1.724
P= 0.011
Survival (Rate)
Time (Months)
C
Lower Expression
Higher Expression
NR4A3
PTGS2
SERPINB8
NR4A2
IL6
AREG
RGS1
EGR1
Dead (P=0.0277)
D
Higher Expression
Lower Expression
EGR1
AREG
NR4A2
NR4A3
PTGS2
IL6
RGS1
SERPINB8
Dead (P=0.011)

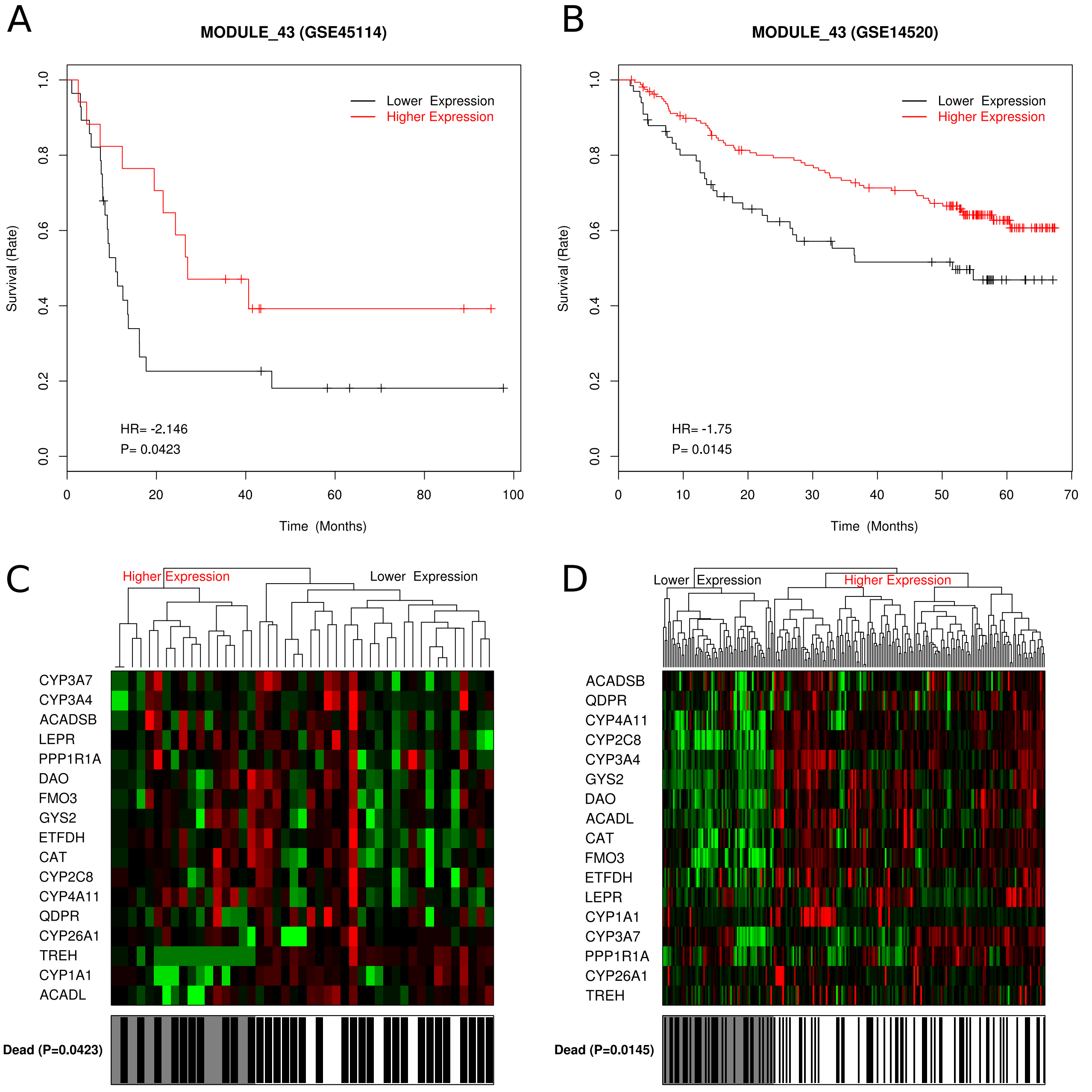
A
MODULE_43 (GSE45114)
Lower Expression
Higher Expression
HR= -2.146
P= 0.0423
Survival (Rate)
Time (Months)
B
MODULE_43 (GSE14520)
Lower Expression
Higher Expression
HR= -1.75
P= 0.0145
Survival (Rate)
Time (Months)
C
Higher Expression
Lower Expression
CYP3A7
CYP3A4
ACADSB
LEPR
PPP1R1A
DAO
FMO3
GYS2
ETFDH
CAT
CYP2C8
CYP4A11
QDPR
CYP26A1
TREH
CYP1A1
ACADL
Dead (P=0.0423)
D
Lower Expression
Higher Expression
ACADSB
QDPR
CYP4A11
CYP2C8
CYP3A4
GYS2
DAO
ACADL
CAT
FMO3
ETFDH
LEPR
CYP1A1
CYP3A7
PPP1R1A
CYP26A1
TREH
Dead (P=0.0145)

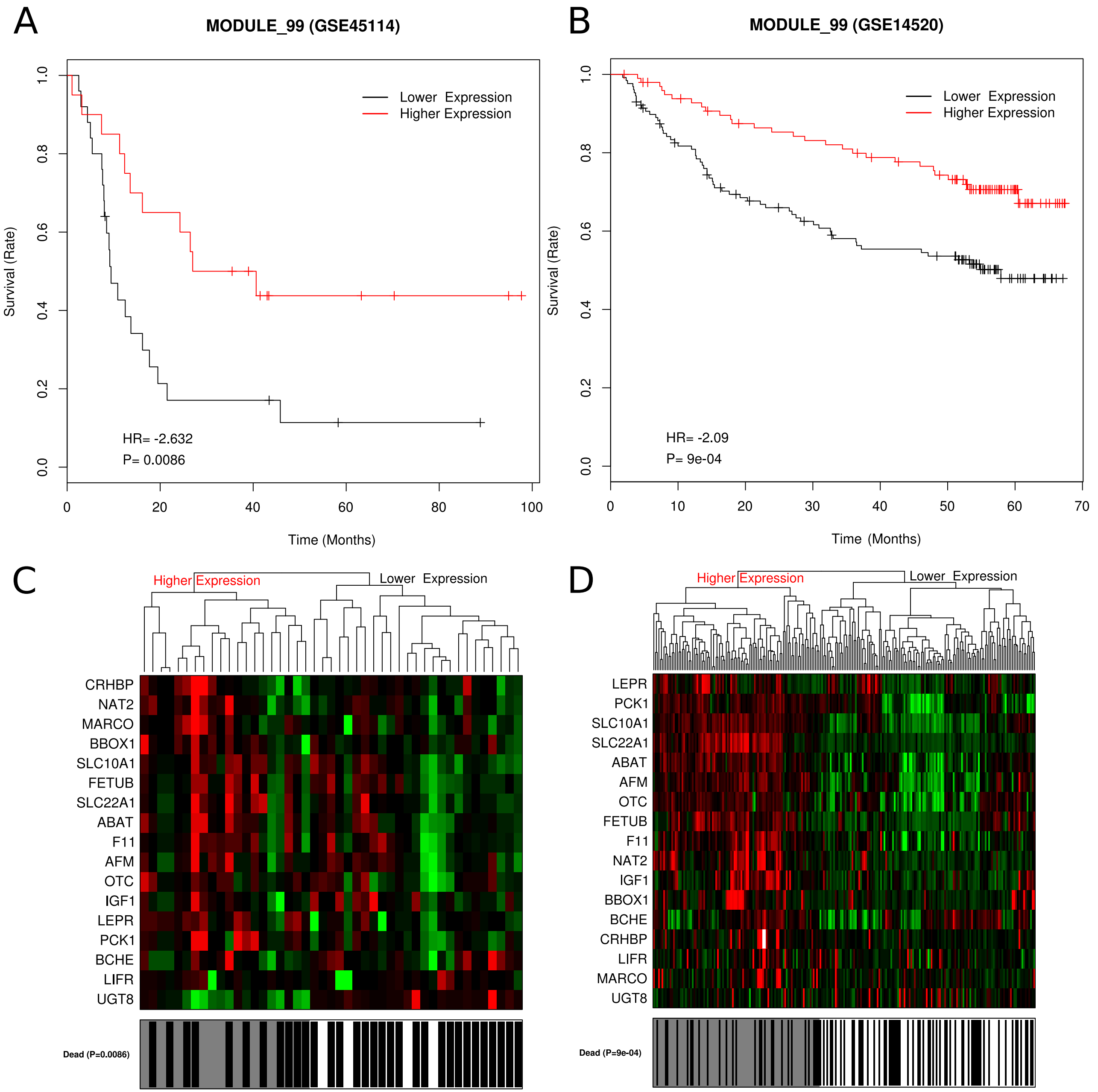
A
MODULE_99 (GSE45114)
Survival (Rate)
Time (Months)
Lower Expression
Higher Expression
HR= -2.632
P= 0.0086
B
MODULE_99 (GSE14520)
Survival (Rate)
Time (Months)
Lower Expression
Higher Expression
HR= -2.09
P= 9e-04
C
Higher Expression
Lower Expression
CRHBP
NAT2
MARCO
BBOX1
SLC10A1
FETUB
SLC22A1
ABAT
F11
AFM
OTC
IGF1
LEPR
PCK1
BCHE
LIFR
UGT8
Dead (P=0.0086)
D
Higher Expression
Lower Expression
LEPR
PCK1
SLC10A1
SLC22A1
ABAT
AFM
OTC
FETUB
F11
NAT2
IGF1
BBOX1
BCHE
CRHBP
LIFR
MARCO
UGT8
Dead (P=9e-04)

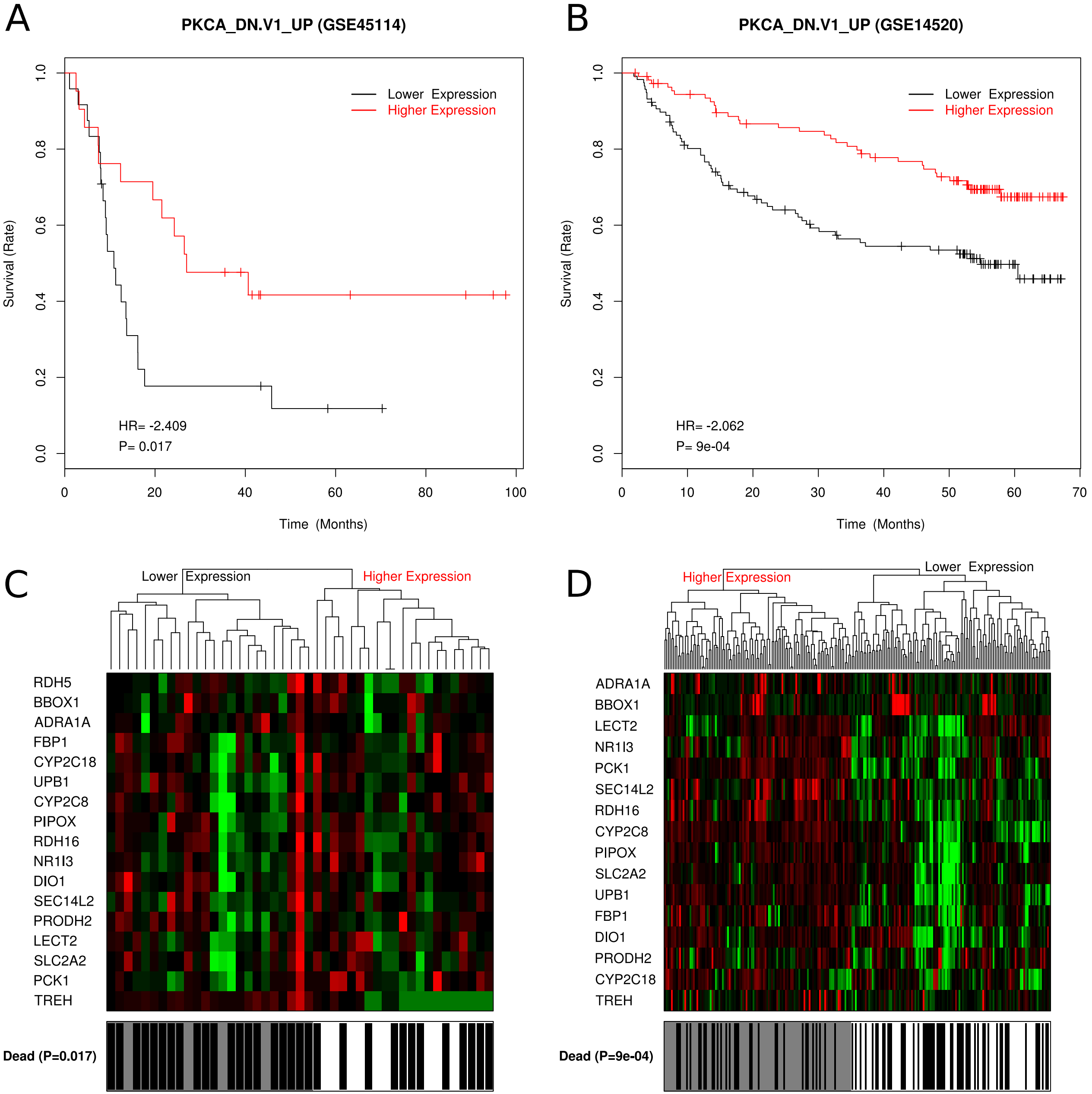

A
PKCA_DN.V1_UP (GSE45114)
Survival (Rate)
Time (Months)
Lower Expression
Higher Expression
HR= -2.409
P= 0.017
B
PKCA_DN.V1_UP (GSE14520)
Survival (Rate)
Time (Months)
Lower Expression
Higher Expression
HR= -2.062
P= 9e-04
C
Lower Expression
Higher Expression
RDH5
BBOX1
ADRA1A
FBP1
CYP2C18
UPB1
CYP2C8
PIPOX
RDH16
NR1I3
DIO1
SEC14L2
PRODH2
LECT2
SLC2A2
PCK1
TREH
Dead (P=0.017)
D
Higher Expression
Lower Expression
ADRA1A
BBOX1
LECT2
NR1I3
PCK1
SEC14L2
RDH16
CYP2C8
PIPOX
SLC2A2
UPB1
FBP1
DIO1
PRODH2
CYP2C18
TREH
Dead (P=9e-04)

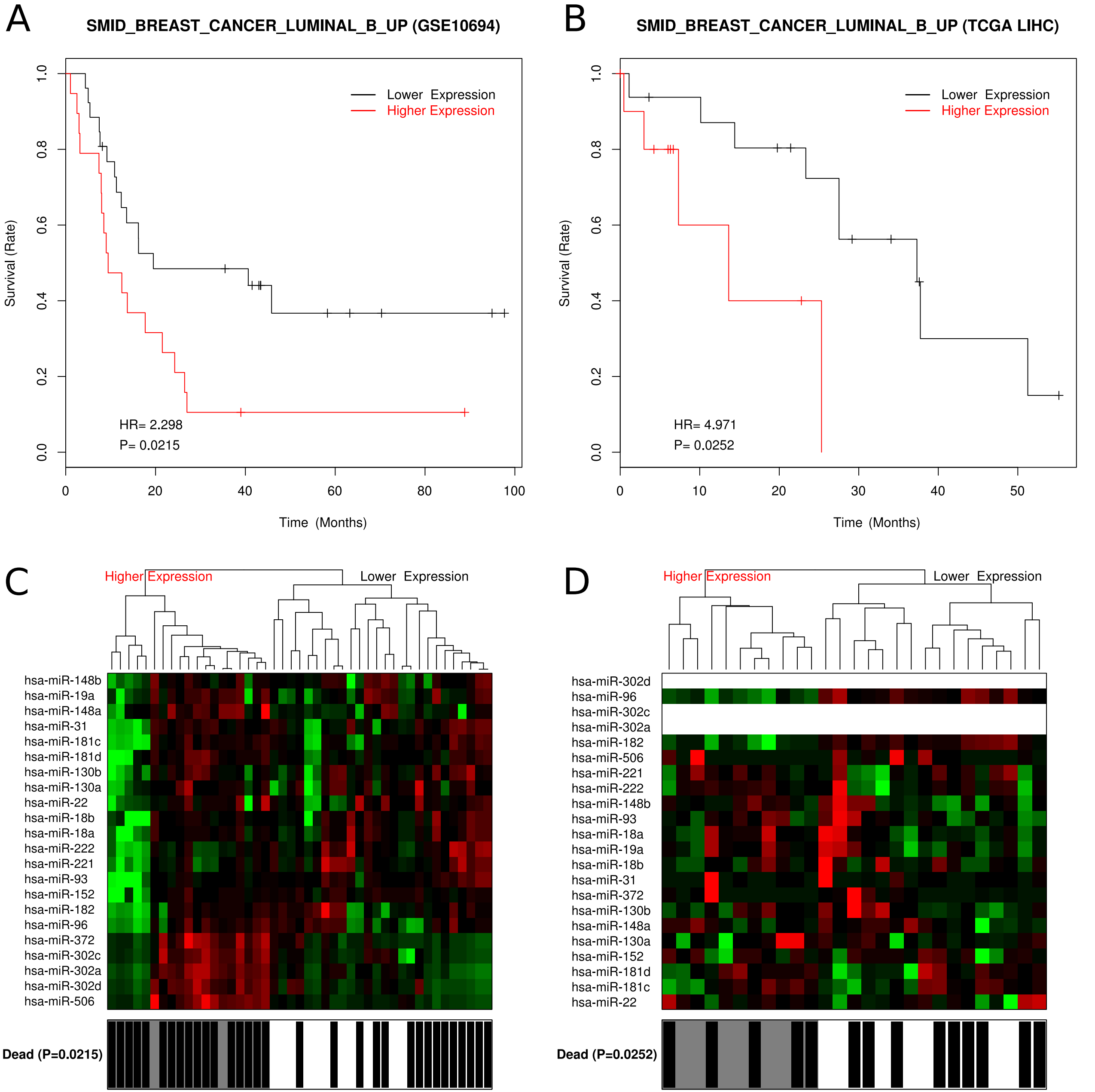
A
SMID_BREAST_CANCER_LUMINAL_B_UP (GSE10694)
Lower Expression
Higher Expression
HR= 2.298
P= 0.0215
Survival (Rate)
Time (Months)
B
SMID_BREAST_CANCER_LUMINAL_B_UP (TCGA LIHC)
Lower Expression
Higher Expression
HR= 4.971
P= 0.0252
Survival (Rate)
Time (Months)
C
Higher Expression
Lower Expression
hsa-miR-148b
hsa-miR-19a
hsa-miR-148a
hsa-miR-31
hsa-miR-181c
hsa-miR-181d
hsa-miR-130b
hsa-miR-130a
hsa-miR-22
hsa-miR-18b
hsa-miR-18a
hsa-miR-222
hsa-miR-221
hsa-miR-93
hsa-miR-152
hsa-miR-182
hsa-miR-96
hsa-miR-372
hsa-miR-302c
hsa-miR-302a
hsa-miR-302d
hsa-miR-506
Dead (P=0.0215)
D
Higher Expression
Lower Expression
hsa-miR-302d
hsa-miR-96
hsa-miR-302c
hsa-miR-302a
hsa-miR-182
hsa-miR-506
hsa-miR-221
hsa-miR-222
hsa-miR-148b
hsa-miR-93
hsa-miR-18a
hsa-miR-19a
hsa-miR-18b
hsa-miR-31
hsa-miR-372
hsa-miR-130b
hsa-miR-148a
hsa-miR-130a
hsa-miR-152
hsa-miR-181d
hsa-miR-181c
hsa-miR-22
Dead (P=0.0252)
